# Induction of TFEB promotes Kupffer cell survival and reduces lipid accumulation in MASLD

**DOI:** 10.1097/HC9.0000000000000853

**Published:** 2025-11-24

**Authors:** Mandy M. Chan, Sabine Daemen, Wandy Beatty, Kathleen Byrnes, Kevin Cho, Daniel Ferguson, Natalie Feldstein, Christopher Park, Zhen Guo, Arick C. Park, Christina F. Fu, Kira L. Florczak, Li He, Bin Q. Yang, Ali Javaheri, Gary J. Patti, Brian N. Finck, Babak Razani, Joel D. Schilling

**Affiliations:** 1 Department of Medicine, Washington University in St. Louis, St. Louis, Missouri, USA; 2 Department of Medicine, Cardiovascular Research Institute Maastricht, Maastricht University Medical Center, Maastricht, The Netherlands; 3 Department of Molecular Microbiology, Washington University in St. Louis, St. Louis, Missouri, USA; 4 Department of Pathology and Immunology, Washington University in St. Louis, St. Louis, Missouri, USA; 5 Department of Chemistry, Siteman Cancer Center, Center for Mass Spectrometry and Metabolic Tracing, Washington University in St. Louis, St. Louis, Missouri, USA; 6 Department of Medicine, Center for Human Nutrition, Washington University in St. Louis, St. Louis, Missouri, USA; 7 Division of Cardiology, Massachusetts General Hospital, Harvard Medical School, Boston, Massachusetts, USA; 8 Vascular Medicine Institute, Department of Medicine, University of Pittsburgh School of Medicine and UPMC, Pittsburgh, Pennsylvania, USA

**Keywords:** de novo lipogenesis, ferroptosis, lipid peroxidation, macrophages, oxidative stress

## Abstract

**Background:**

Kupffer cells (KCs) are the tissue-resident macrophages of the liver, where they serve a critical role in maintaining liver tissue homeostasis and act as a filter for circulation. The composition of hepatic macrophages changes during metabolic dysfunction–associated liver disease (MASLD), with the loss of resident KCs being a hallmark of disease progression. The mechanism(s) and consequences of KC death in metabolic liver disease have yet to be defined. Transcription factor EB (TFEB) is a master regulator of lysosome function and lipid metabolism, which has been shown to protect macrophages from lipid stress in atherosclerosis. We hypothesized that TFEB would improve KC fitness in MASLD.

**Methods:**

To investigate the potential beneficial effect of TFEB induction in KCs, we created a transgenic mouse in which TFEB was overexpressed specifically in KCs and evaluated its impact on disease pathogenesis in high-fat, high-sucrose (HFHS) and choline-deficient diet models of MASLD.

**Results:**

We found that TFEB induction protected KCs from cell death in both models of MASLD. KC preservation through TFEB induction reduced liver steatosis with HFHS diet via mechanisms that were dependent on macrophage lysosomal lipolysis and mitochondrial fatty acid oxidation. Fibrosis was unchanged in choline-deficient diet studies. TFEB protected KCs from cell death by diminishing oxidative stress and reducing ferroptosis through a mechanism that involved enhanced NADPH levels.

**Conclusions:**

TFEB induction promotes KC fitness upon lipid stress during MASLD. Preservation of lipid-adapted KCs demonstrates beneficial effects against liver steatosis and protects portal filtration during MASLD.

## Graphical abstract

**Figure:**
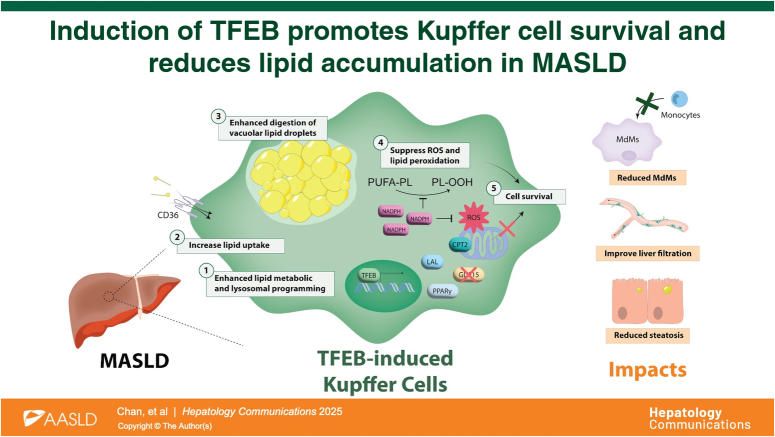

## INTRODUCTION

Metabolic dysfunction–associated steatotic liver disease (MASLD) is characterized by excessive fat accumulation in the liver. MASLD is associated with hepatic injury, inflammation, and fibrosis, at which point it evolves into the more severe form known as metabolic dysfunction–associated steatohepatitis (MASH).^1^ MASLD is the most common form of chronic liver disease, affecting one-third of the population in the Western hemisphere, and MASH-associated cirrhosis is the leading cause of liver transplantation.^2^ Metabolic liver disease is intimately linked with obesity and type 2 diabetes (T2D), and nearly 75% of people with obesity and T2D have MASLD.^3^ Although the FDA recently approved Rezdiffra for the treatment of MASH, only ~25% of patients treated showed an improvement in liver fibrosis.^4^ Therefore, it remains critical to elucidate the cellular and molecular determinants that promote MASLD progression.

Kupffer cells (KCs) are embryonically derived, liver-resident macrophages that account for >90% of the macrophages in a non-diseased liver.^5^ KCs reside in the lumen of liver sinusoids with a greater periportal density.^6^ These resident macrophages filter the bloodstream, modulate immune activation, ingest dead cells, and sequester excessive iron.^7^^–^^10^ In a normal liver, KCs maintain their cell number by self-renewal; however, loss of KCs by genetic depletion,^11^^,^^12^ clodronate-containing liposomes,^6^ or irradiation^13^ leads to rapid infiltration of monocytes into the liver, where they differentiate into monocyte-derived macrophages (MdMs), some of which replace KCs.^11^^–^^13^ Recent single-cell RNA sequencing, multimarker flow cytometry, and spatial imaging have revealed significant heterogeneity and dynamic changes in the composition of liver macrophages in human and mouse MASLD.^14^^,^^15^ In addition to F4/80 and CD11b, KCs express TIM4, MARCO, CD163, CLEC4F, and VSIG4 that distinguish them from other disease-associated MdMs. Furthermore, we and others have demonstrated that the number of *bona fide* KCs decreases in mouse models of MASLD.^16^^–^^18^ The mechanisms accounting for the loss of resident KCs are not known, but recent reports have linked lysosomal stress and iron accumulation to KC death during MASLD.^19^^,^^20^

Transcription factor EB (TFEB) is a member of the MiT transcription factor family and is a master regulator of lipid metabolism, lysosomal biogenesis, and autophagy.^21^ Activation of TFEB may be a therapeutic approach for diseases associated with lysosomal dysfunction, including neurodegenerative diseases^22^ and atherosclerosis.^23^^,^^24^ Genetic overexpression and pharmacological activation of TFEB in macrophages have been shown to (1) rescue lipid-induced lysosome dysfunction in atherosclerosis and (2) protect against obesity via growth differentiation factor 15 (GDF15) expression.^23^^,^^25^ Given the documented advantages of TFEB in macrophages, we hypothesized that stimulating this pathway in KCs may confer protective effects during MASLD. To explore the impact of TFEB activation in KCs, we generated mice in which TFEB was overexpressed in a KC-specific manner and assessed the phenotype of these mice on two distinct MASH diets. We discovered that TFEB-overexpression rescues KC death *in vivo*, augments their lipid uptake and metabolism, and reduces liver steatosis after MASLD induction.

## METHODS

### Mice

Clec4f^Cre^ and Flox/stop/flox-TFEB-3xFLAG transgenic mice were obtained from Dr Charlotte Scott^11^ and Dr Andrea Ballabio,^21^ respectively. KC-TdT reporter mice were bred in-house by crossing Clec4f^Cre^ mice with ROSA26^flox/stop/flox^-TdTomato mice. LysM^Cre^ and TFEB^fl/fl^ mice were obtained from Dr Babak Razani. CPT2^fl/fl^ mice were obtained from Dr Jennifer Philips. LAL^flox/flox^ mice were obtained from Dr Ali Javaheri. Cx3Cr1^CreER^–ROSA26^flox/stop/flox-TdT^ mice were obtained from Dr Kory Lavine. GDF15^fl/fl^ mice were generated by the CRISPR–Cas9 system to insert flox/stop/flox cassettes around the second exon. Wild-type male C57BL/6 animals were purchased from Jackson Laboratory. All mice were bred in-house at Washington University School of Medicine, St. Louis. High-fat, high-sucrose diet (HFHS) (42% kcal fat diet with increased sucrose and 1.25% cholesterol) was purchased from Envigo Teklad (Cat# TD.120528). Choline-deficient, amino acid-defined diet (CDAA) was purchased from Research Diets (Cat# A06071309). All animals were fed *ad libitum* with unlimited access to drinking water. All animal protocols were approved by the Institutional Animal Care and Use Committee at Washington University School of Medicine. Additional information is described in the Supplemental Methods, http://links.lww.com/HC9/C188 including qPCR primer sequences (Supplementary Table S2).

### Cell culture

To generate BMDMs, bone marrow was isolated from the femur and tibia of mice and differentiated with M-CSF-containing condition media as described in Supplemental Methods, http://links.lww.com/HC9/C188. For flow cytometry-based experiments, cells were plated on suspension plates at 5 × 10^5^ cells/mL; for all other assays, cells were plated on tissue culture-treated plates at the same density.

For primary KC culture to obtain RNA and NADP^+^/NADPH assay, livers were perfused with collagenase as described in the Supplemental Methods, http://links.lww.com/HC9/C188. After differential centrifugation, CD45^+^ cells were positively selected using CD45 microbeads and MS columns (Miltenyi Biotec, Cat# 130-052-301; #130-042-201). CD45^+^ cells from 1 mouse were plated onto tissue culture plates for 2 hours of incubation at 37 °C to allow adhesion. Non-adherent cells were then washed off with PBS twice, and adherent cells were used.

## RESULTS

### Induction of TFEB in KCs alters macrophage lipid-handling

**FIGURE 1:**
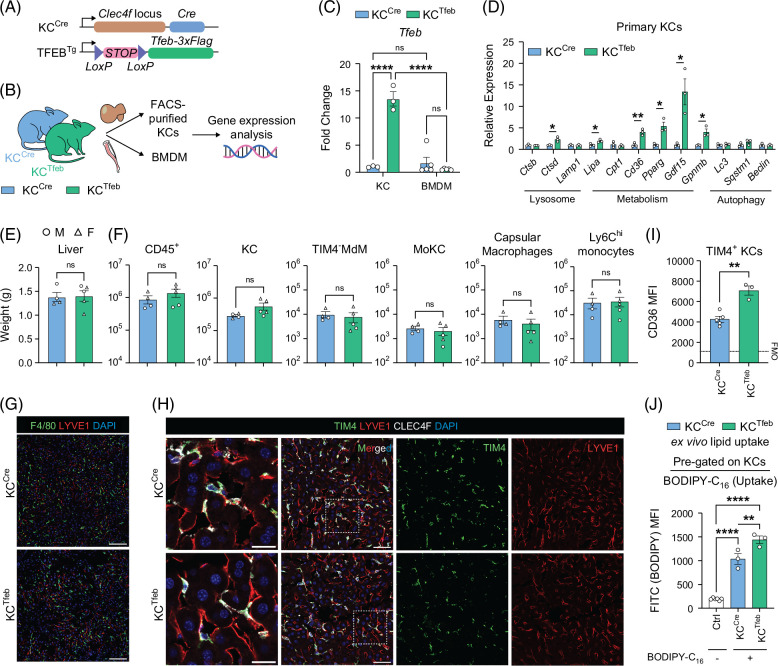
Overexpression of TFEB in KCs enhances lipid uptake and metabolic gene expression. (A) Constructs to generate KC-specific TFEB-overexpressing mice, or KC^Tfeb^. (B) Schematics for isolating KCs and BMDMs from KC^Cre^ and KC^Tfeb^ animals for gene expression analyses. (C) Gene expression of *Tfeb* in KCs and BMDMs isolated from KC^Cre^ and KC^Tfeb^ mice (n=3/group; technical duplicate for BMDMs). (D) Gene expression of selected targets of TFEB in WT-KCs or TFEB-KCs (n=3/group). (E–H) Characteristics of 8–10-week-old KC^Cre^ and KC^Tfeb^ mice. (E) Liver weights and (F) flow cytometric quantification of different macrophage and monocyte populations (n=4–5/group). Circles represent males; triangles represent females. (G) Representative immunofluorescence images of livers showing overall macrophage distribution. Green: F4/80; red: LYVE1; blue: DAPI. Scale bar=100 µm. (H) Representative immunofluorescence images of livers showing KC-specific markers with LSEC-lined sinusoids. Green: TIM4; red: LYVE1; white: CLEC4F; blue: DAPI. Scale bar=50 µm. Insert scale bar=20 µm. (I) Mean fluorescent intensity (MFI) of CD36 in WT or TFEB-KCs quantified by flow cytometry (n=3–5/group). (J) MFI of BODIPY-C_16_ signal in WT or TFEB-KCs quantified by flow cytometry (n=3/group). Ctrl=no BODIPY-C_16_ controls. Data represent individual biological replicates and are presented as means±SEM. *p*-values were calculated using (C) 2-way ANOVA, followed by multiple *t* tests, (D–F, I) unpaired 2-tailed Student *t* tests, and (J) one-way ANOVA followed by multiple *t* tests. NS=not significant, *p<0.05, **p<0.01, ***p<0.001, and ****p<0.0001. Abbreviations: BMDMs, bone marrow–derived macrophages; FMO, Fluorescence minus one; KC, Kupffer cell; TFEB, transcription factor EB; WT, wild type.

Enhancing TFEB expression or activity in macrophages has been shown to attenuate lipotoxic cell death and reduce atherosclerosis in mice.^24^^,^^26^ To investigate the impact of TFEB on KCs during MASLD, we generated KC-specific TFEB-overexpressing mice (KC^Tfeb^) by crossing the Clec4f^Cre^ line (KC^Cre^)^11^ with mice containing a flox/stop/flox cassette followed by a *Tfeb*–3×FLAG transgene^21^ (Figure 1A). *Tfeb* mRNA levels were increased in purified KCs from KC^Tfeb^ mice but not in BMDMs from the same mice (Figures 1B, C). TFEB-overexpressing KCs had increased expression of genes related to lysosome function and lipid metabolism, whereas the mRNA levels of autophagic genes were not impacted (Figure 1D).

To assess Cre expression in MASLD, we crossed these animals to *Rosa26* ^flox/stop/flox^ TdTomato (TdT) reporter mice (KC^Cre-TdT^ and KC^Tfeb-TdT^). After 16 weeks of a high-fat, high-sucrose, and high-cholesterol (HFHS) diet, over 80% of TIM4^+^VSIG4^+^ KCs were TdT^+^ (Supplemental Figures S1A, B, http://links.lww.com/HC9/C188). In MASLD, TIM4^lo^ macrophages consist of monocyte-derived KCs (MoKCs), which express VSIG4/CLEC4F, and lipid-associated macrophages (LAMs)/pre-MoKCs termed “non-KCs” here, which are F4/80^hi^ but lack both TIM4 and VSIG4 expression and frequently express *Trem2* (Supplemental Figure S1A, http://links.lww.com/HC9/C188).^14^^,^^16^^,^^17^ We found that 60%–80% of MoKCs (TIM4^−^VSIG4^+^) were TdT^+^, but non-KCs (TIM4^−^VSIG4^−^) and monocytes were mostly reporter-negative (Supplemental Figure S1B, http://links.lww.com/HC9/C188). CLEC2 is expressed by macrophages early after entering the liver and is present in KCs, MoKCs, and non-KCs regardless of TFEB-overexpression (Supplemental Figure S1C, http://links.lww.com/HC9/C188).

At baseline, transgenic mice had similar liver weight and KC number compared with control mice (Figures 1E, F and Supplemental Figure S1A, http://links.lww.com/HC9/C188). There were no differences in the sinusoidal localization, morphology, and cell surface marker expression in KCs between the genotypes (Figures 1G, H). Overexpression of TFEB led to increased expression of CD36 and enhanced fatty acid (FA) uptake in KCs (Figures 1D, I, J). However, micropinocytosis and lysosomal number/activity were similar between WT and TFEB-KCs at baseline (Supplemental Figures S1C, D, http://links.lww.com/HC9/C188), which may reflect the high baseline lysosomal activity of KCs. Thus, we conclude that TFEB overexpression in KCs promotes the uptake of FA without affecting the baseline number and composition of macrophages in the liver.

### KC-specific TFEB induction preserves KC number and reduces MdM infiltration

**FIGURE 2:**
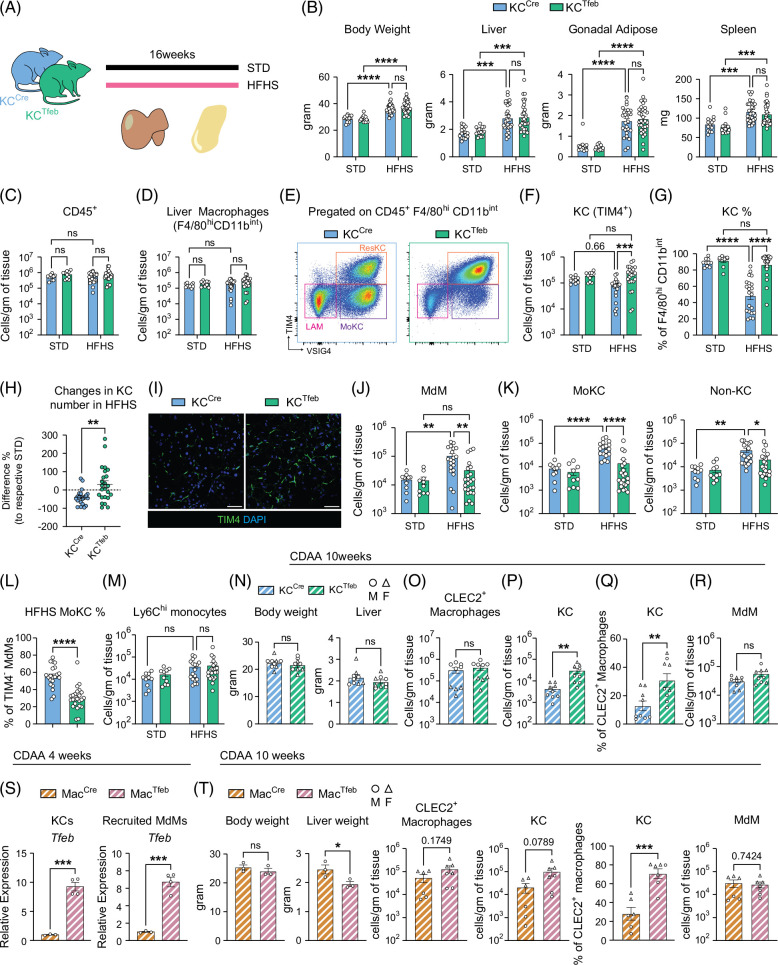
TFEB induction maintains KC numbers and reduces MdM accumulation in MASLD. (A–M) STD versus HFHS diet experiments with 8–10-week-old male KC^Cre^ and KC^Tfeb^ littermate mice (n=10–24/group). (A) Schematic of experiment. (B) Final body and organ weights of STD-fed or HFHS diet-fed mice. (C) Flow cytometric quantification of live, single CD45^+^ cells, (D) total liver macrophages (CD45^+^F4/80^hi^CD11b^int^). (E) Representative flow plots of liver macrophages in HFHS livers. (F) Flow cytometric quantification of KCs (TIM4^+^). (G) Percentage of KCs within total liver macrophages. (H) Changes in KC numbers comparing HFHS-fed KC^Cre^ and KC^Tfeb^ littermates with respective STD-fed mice. (I) Representative immunofluorescence images of the livers of HFHS diet-fed mice stained with TIM4 to identify KCs. Green: TIM4; blue: DAPI. Scale bar=50 µm. (J) Flow cytometric quantification of MdMs (TIM4^−^) and (K) MdM subsets, MoKCs (TIM4^−^VSIG4^+^), and LAMs (TIM4^−^VSIG4^−^). (L) Percentages of MoKCs among total TIM4^−^ MdMs. (M) Flow cytometric quantification of Ly6C^hi^ monocytes (F4/80^lo^CD11b^+^MHCII^−^Ly6C^+^). (N–R) KC^Cre^ and KC^Tfeb^ littermates were fed a 10-week fibrogenic CDAA diet (n=9/group). Circles represent males and triangles represent females. (N) Final body and liver weight. (O) Flow cytometric quantification of CLEC2^+^ liver macrophages (CLEC2^+^F4/80^hi^CD11b^int^) and (P) KCs (TIM4^+^). (Q) Percentage of KCs in CLEC2^+^ macrophages. (R) Quantification of MdMs (CLEC2^+^TIM4^−^). (S) Relative expression of *Tfeb* in FACS-purified KCs (TIM4^+^VSIG4^+^) recruited MdMs (CD45^+^F480^hi^ CD11b^hi/int^ MHCII^+^ Ly6C^−^ CLEC2^+/−^CD11c^−^TIM4^−^VSIG4^−^) flow-sorted from 4-week CDAA diet-fed mice (n=3–4/group). (T) Body weights, liver weights, quantification of KC number and percentages, and quantification of MdMs in Mac^Cre^ and Mac^Tfeb^ littermates fed 10 weeks of CDAA diet (n=6-7/group). Circles represent males and triangles represent females. Data represent individual biological replicates and are presented as means±SEM. *p*-values were calculated using (B–D, F, G, J-K, M) 2-way ANOVA followed by multiple *t* tests and (H, L, N–T) 2-tailed unpaired *t* tests. NS=not significant, *p<0.05, **p<0.01, ***p<0.001, and ****p<0.0001. Abbreviations: CDAA, choline-deficient amino acid-defined; HFHS, high-fat, high-sucrose, and high-cholesterol; KC, Kupffer cell; LAM, lipid-associated macrophages; MdM, monocyte-derived macrophage; MoKCs, monocyte-derived KCs; MASLD, metabolic dysfunction–associated steatotic liver diseases; STD, standard diet; TFEB, transcription factor EB.

To assess the impact of TFEB in KCs on MASLD pathogenesis, we placed KC^Cre^ and KC^Tfeb^ mice on the HFHS diet for 16 weeks (Figure 2A). Male mice of both genotypes gained similar degrees of weight with comparable increases in liver, gonadal adipose tissue, and spleen size (Figure 2B). Female mice did not gain as much weight, but again, there was no difference between the genotypes (Supplemental Figure S2A, http://links.lww.com/HC9/C188). The degree of glucose intolerance was also similar between the genotypes (Supplemental Figure S2B, http://links.lww.com/HC9/C188).

To determine the effect of TFEB on macrophage numbers, flow cytometry was performed on the livers of male KC^Cre^ and KC^Tfeb^ mice after HFHS diet. Neither diet nor genotype changed the number of CD45^+^ immune cells or liver macrophages (Figures 2C, D). However, TIM4^hi^ KC number decreased in control mice on HFHS diet, but not in KC^Tfeb^ mice (Figures 2E–I). KC^Tfeb^ mice also had fewer MdMs in the liver following the HFHS diet (Figures 2E, J). Although both MoKCs and non-KCs were decreased in the KC^Tfeb^ livers, MoKCs were more dramatically reduced (Figures 2K, L). The number of Ly6C^hi^ monocytes and lymphocyte populations also did not differ significantly between the genotypes (Figure 2M and Supplemental Figure S2C, http://links.lww.com/HC9/C188). The expression of *Il1b* was decreased in liver tissue, while *Ccl2* transcript was similar between genotypes (Supplemental Figure S2D, http://links.lww.com/HC9/C188).

Steatosis drives infiltration of MdMs to the liver independent of KC loss.^27^ To determine whether TFEB in KCs impacts the early stage of MdM recruitment, we placed KC^Cre^ and KC^Tfeb^ mice on a short-term (8-week) HFHS diet (Supplemental Figure S2E, http://links.lww.com/HC9/C188). Mature KC numbers were preserved in both WT and transgenic mice; however, the number of mature MdMs was still decreased in KC^Tfeb^ livers (Supplemental Figures S2F–J, http://links.lww.com/HC9/C188). Liver *Il1b*, but not *Ccl2*, was again significantly lowered in KC^Tfeb^ livers at this early stage (Supplemental Figure S2K, http://links.lww.com/HC9/C188). Similar findings were seen in female mice, even though they did not gain as much weight as their male counterparts (Supplemental Figures S2L–Q, http://links.lww.com/HC9/C188).

We also confirmed that TFEB protects KCs in mice fed a choline-deficient amino acid-defined (CDAA) diet for 10 weeks to induce MASH (Figures 2N–Q). CDAA diet induces robust infiltration of monocytes and immature MdMs; therefore, CLEC2 expression was used to identify more mature liver macrophages^18^ (Supplemental Figure S2R, http://links.lww.com/HC9/C188). MdM number markedly increased with the CDAA diet and was similar between the genotypes (Figure 2R). To ensure the robustness of this observation, we repeated this experiment with mice with LysM–Cre (Mac^Cre^) crossed to the TFEB transgene (Mac^Tfeb^). TFEB was overexpressed by 5-10-fold in KCs and recruited MdMs isolated from Mac^Tfeb^ mice (Figure 2S). Again, the KC number was higher in Mac^Tfeb^ mice after CDAA diet feeding (Figure 2T). Thus, we conclude that TFEB induction maintains resident KC numbers in MASH.

### TFEB induction in KCs alters the hepatic lipid landscape in MASLD

**FIGURE 3:**
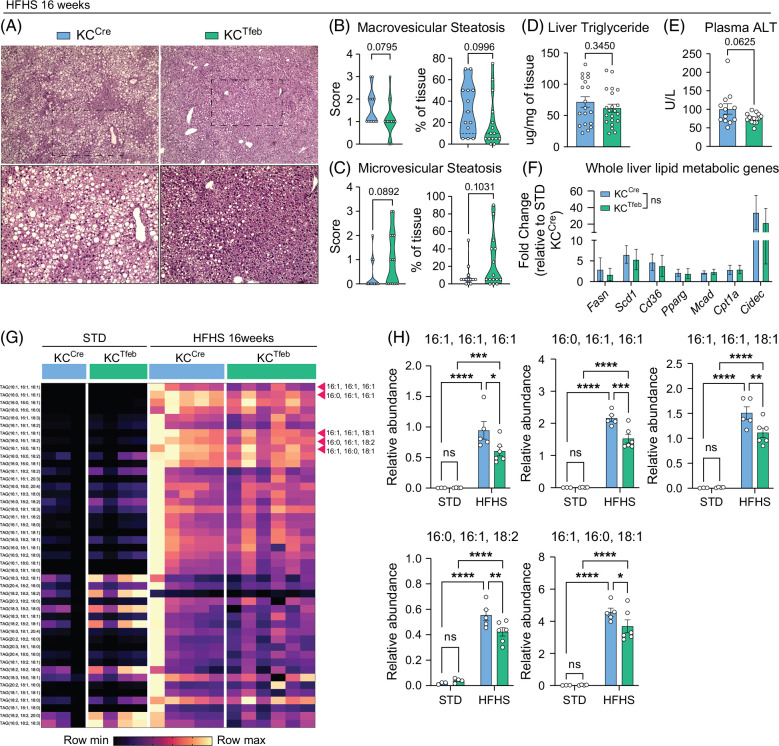
TFEB induction in KCs reduces hepatic steatosis. (A–D) Liver steatosis phenotyping for 16-week HFHS diet-fed KC^Cre^ and KC^Tfeb^ mice (n=13–21/group). (A) Representative H&E images of livers. (B) Macrovesicular and (C) microvesicular steatosis scores and percentages as determined by a blinded pathologist. (D) Liver TAG measurement. (E) Plasma ALT measurement from 16-week HFHS diet-fed KC^Cre^ and KC^Tfeb^ mice (n=12–15/group). (F) qPCR gene expression analyses of selected lipid metabolic genes in whole liver tissues of KC^Cre^ and KC^Tfeb^ mice after 16 weeks of STD or HFHS feeding (n=5–7/group). Shown are fold changes relative to STD-fed KC^Cre^. (G) Heatmap of normalized (within individual TAG species) relative abundance of 45 TAG species from hepatic tissue, accompanied by quantification of selected species with statistical significance (n=3–6/group). Data represent individual biological replicates and are presented as means±SEM. *p-*values were calculated using (B–F) unpaired 2-tailed Student *t* tests, and (H) 2-way ANOVA followed by multiple *t* tests. NS=not significant, *p<0.05, **p<0.01, ***p<0.001, and ****p<0.0001. Abbreviations: HFHS, high-fat, high-sucrose, and high-cholesterol; H&E, hematoxylin and eosin; KCs, Kupffer cells; qPCR, quantitative polymerase chain reaction; STD, standard diet; TAG, triglyceride; TFEB, transcription factor EB.

We next investigated the impact of TFEB induction in KCs on liver steatosis. H&E sections were scored by a blinded pathologist and revealed that KC^Tfeb^ mice had a reduction in macrovesicular, but not microvesicular, steatosis in the liver (Figures 3A–C). Liver triglyceride (TAG) and plasma alanine transaminase (ALT) levels were also modestly reduced in the KC^Tfeb^ mice while liver cholesterol and plasma triglyceride concentration remained unchanged (Figures 3D, E and Supplemental Figure S3A, http://links.lww.com/HC9/C188). Interestingly, expression of genes related to de novo lipogenesis or fatty acid oxidation (FAO) in whole liver was altered by HFHS diet, but no difference was seen between genotypes (Figure 3F). Thus, changes in hepatocyte metabolism were unlikely to explain the lipid differences. Using targeted lipidomics, we found a significant increase in TAG species in livers after the HFHS diet (Figure 3G). Among the TAG species measured, several were significantly reduced in the KC^Tfeb^ livers (Figures 3G, H and Supplemental Figure S3B, http://links.lww.com/HC9/C188). Livers from KC^Tfeb^ mice also had increased levels of low-abundance TAG containing unsaturated 18-carbon chains at baseline (Supplemental Figure S3C, http://links.lww.com/HC9/C188). The extent of fibrosis in the HFHS diet was minimal (Supplemental Figures S3D, E, http://links.lww.com/HC9/C188). Therefore, we performed picrosirius red (PSR) imaging on the livers of KC^Cre^ and KC^Tfeb^ mice after 10 weeks of the fibrogenic CDAA diet. The average extent of fibrosis by PSR quantification was similar between the genotypes (Supplemental Figure 3F, http://links.lww.com/HC9/C188). This finding aligns with the fact that both KC^Cre^ and KC^Tfeb^ mice have a similar degree of MdM influx with this highly inflammatory diet.

### MASLD induces lysosomal, metabolic, and pro-survival transcriptomic signatures in TFEB-overexpressing KCs

**FIGURE 4:**
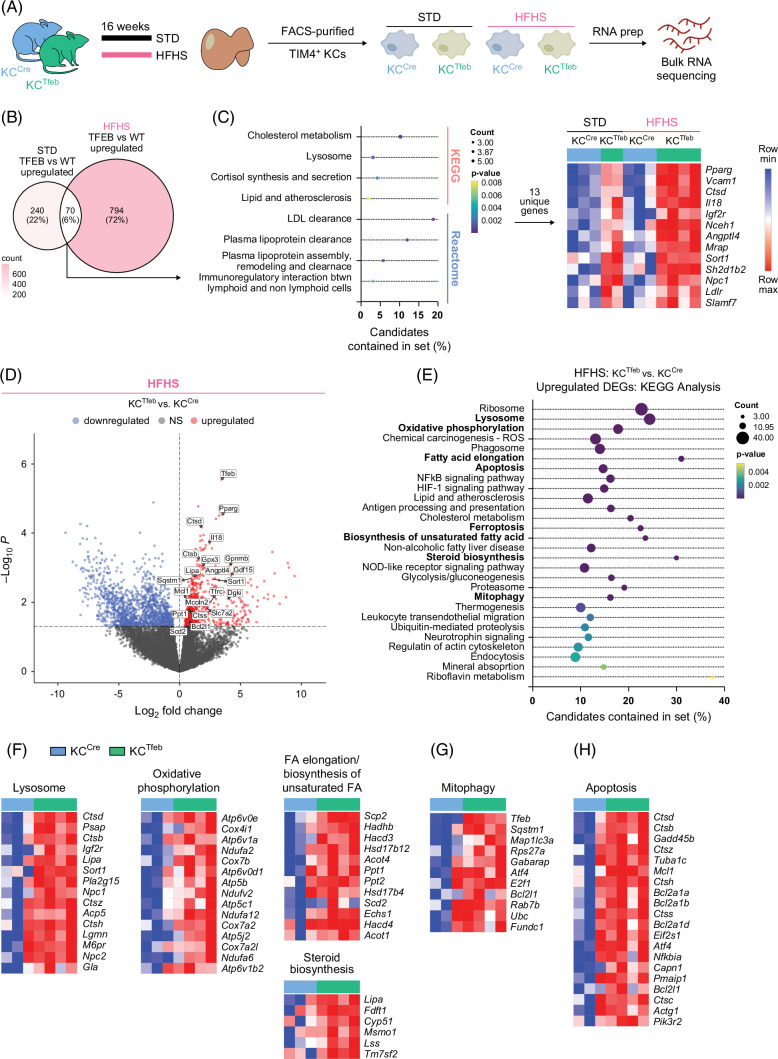
MASLD augments TFEB-mediated lysosomal and lipid metabolic transcriptional programs in KCs. (A) Schematic of experiments to isolate KCs from different conditions for bulk RNAseq (n=2–4/group). (B, C) (B) Venn diagram comparing significantly upregulated DEGs and (C) KEGG and Reactome pathway analyses of the 70 commonly upregulated genes, among which 13 unique genes contributed to the pathway analyses in HFHS TFEB-KCs versus WT-KCs and STD TFEB-KCs versus WT-KCs. (D–F) (D) Volcano plots, (E) KEGG pathways analysis, (F–H) heatmaps of DEGs in TFEB-KCs versus WT-KCs after HFHS feeding. Log_2_fold change >0, *p*-value <0.05. Abbreviations: DEG, differentially expressed gene; HFHS, high-fat, high-sucrose, and high-cholesterol; KCs, Kupffer cells; KEGG, Kyoto Encyclopedia of Genes and Genomes; MASLD, metabolic dysfunction–associated steatotic liver diseases; STD, standard diet; TFEB, transcription factor EB; WT, wild type.

To assess the impact of TFEB on KC function, we performed bulk RNA sequencing on flow-sorted TIM4^+^ KCs from KC^Cre^ and KC^Tfeb^ animals fed STD or HFHS diet (Figure 4A and Supplemental Table S1, http://links.lww.com/HC9/C189). With the STD diet, TFEB induction in KCs led to upregulation of genes related to cholesterol and lipoic acid metabolism (Supplemental Figures S4A, B, http://links.lww.com/HC9/C188). After the HFHS diet, several upregulated differentially expressed genes (DEGs) in WT-KCs mapped to the PPAR signaling pathway, while downregulated DEGs linked to IL-17 and MAPK signaling pathways (Supplemental Figures S4C–E, http://links.lww.com/HC9/C188). In TFEB-KCs, HFHS diet instead led to the upregulation of lysosomal and PPAR signaling-related pathways as well as downregulation of amino acid metabolism (Supplemental Figures S4F, G, http://links.lww.com/HC9/C188). To discern the effects of TFEB and the diet on KC phenotypes, we compared the significantly upregulated genes in TFEB-KCs in STD and HFHS conditions and identified 70 diet-independent TFEB-modulated genes (Figures 4B, C). Outside of the core TFEB-modulated genes, 794 genes were uniquely expressed by HFHS TFEB-KCs versus 240 by STD TFEB-KCs compared with their WT controls (Figure 4B). The upregulated DEGs in TFEB-KCs included genes such as *Ctsb*, *Ctss*, *Mcoln2*, *Lipa*, *Gdf15*, *Scd2*, and *Sqstm1* (Figure 4D). KEGG pathway analysis of these upregulated genes indicated enrichment in metabolic pathways such as lysosome, oxidative phosphorylation, fatty acid elongation, steroid biosynthesis, and mitophagy (Figures 4D–G). KC2s are a subset of KCs that are purported to have CD36-mediated metabolic function.^28^ In our RNAseq data, most of the KC2-associated transcripts, besides *Cd36*, were either unchanged or downregulated in HFHS TFEB-KCs (Supplemental Figure S4F, http://links.lww.com/HC9/C188). Recently, it was reported that following acute liver injury, a subpopulation of KCs acquires LAM-like features, including the expression of TREM2 and CD36.^29^ Immunofluorescence imaging of TREM2 in HFHS-fed KC^Cre^ and KC^Tfeb^ mice revealed a small subset of CLEC4F^+^TREM2^+^ macrophages; however, there were no differences between genotypes (Supplemental Figure S4I, http://links.lww.com/HC9/C188). CD36 expression was also significantly increased in TFEB-KCs at baseline (Figure 1I) and after MASH diets (Supplemental Figure S4J, http://links.lww.com/HC9/C188). Thus, TFEB induction in KCs enhances pathways related to lipid metabolism and lysosome function during metabolic stress.

### TFEB activates lipid metabolic pathways in KCs

**FIGURE 5:**
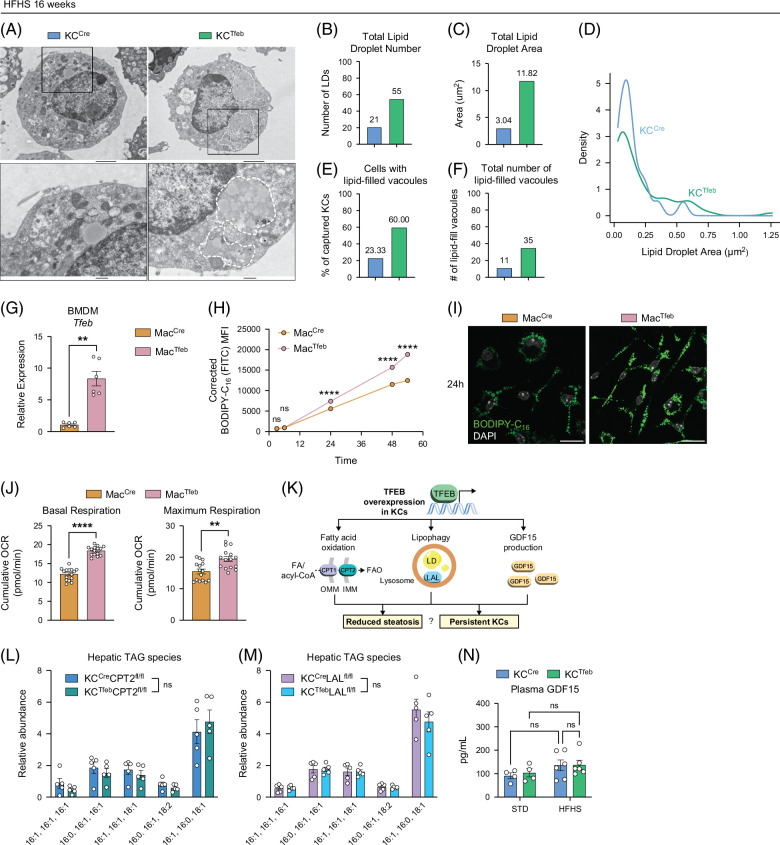
TFEB promotes lipid digestion and oxidative phosphorylation in macrophages. (A–F) FACS-purified KCs from HFHS diet-fed KC^Cre^ and KC^Tfeb^ mice were subjected to electron microscopy (n=2–3/genotype). (A) Representative electron microscopy images of KCs. Top scale bar=2 µm; bottom scale bar=500 nm. (B) Quantification of the total number and (C) total area of LDs across 30 cells per genotype on HFHS diet. (D) Size distribution of LDs per genotype on HFHS diet. (E) Percentage and (F) count of KCs with lipid-filled vacuoles. (G–J) In vitro experiments using BMDMs derived from Mac^Cre^ and Mac^Tfeb^ mice. (G) qPCR gene expression analysis on *Tfeb* (n=6/group). (H) MFI corrected for baseline fluorescence and (I) representative immunofluorescence images of BMDMs cultured with 250  µM oleic acid conjugated to BSA + 1 µM BODIPY-C_16_. Green: BODIPY-C_16_. White: DAPI. Scale bar=20 µm. (J) Cumulative oxygen consumption rate (OCR) for basal and maximum respiration of BMDMs. (K) A graphic representation of potential pathways that TFEB induction could alter in lipid handling. (L–N) FAO, lysosomal lipolysis, and GDF15-deficient KC^Cre^ or KC^Tfeb^ mice were fed an HFHS diet for 16 weeks. (L, M) Hepatic TAG species of KC^Cre^CPT2^fl/fl^, KC^Tfeb^CPT2^fl/fl^, KC^Cre^LAL^fl/fl^, and KC^Tfeb^LAL^fl/fl^ mice (n=5/group). (N) Plasma GDF15 in KC^Cre^GDF15^fl/fl^, KC^Tfeb^GDF15^fl/fl^ mice (n=4–6/group). Data represent (G, L–N) individual biological, or (H–J) technical replicates, and are presented as (B–F) cumulative data or (G, H, J, L, M) means±SEM. *p-*values were calculated using (G) paired and (H, J, L–N) unpaired 2-tailed Student *t* tests. NS=not significant, *p<0.05, **p<0.01, and ****p<0.0001. Abbreviations: BMDMs, bone marrow-derived macrophages; BSA, bovine serum albumin; FACS, fluorescence-activated cell sorting; FAO, fatty acid oxidation; HFHS, high-fat, high-sucrose, and high-cholesterol; KCs, Kupffer cells; LP, lipid droplet; MFI, mean fluorescent intensity; qPCR, quantitative polymerase chain reaction; TAG, triglyceride; TFEB, transcription factor EB.

KCs from WT mice with liver steatosis have enhanced FA uptake compared with STD-fed mice (Supplemental Figure S5A, http://links.lww.com/HC9/C188); however, they have fewer lipid droplets (Supplemental Figures S5B–E, http://links.lww.com/HC9/C188).^27^ Based on the RNAseq data, we hypothesized that altered lipid metabolism in KCs may reduce liver steatosis in fatty liver disease. Therefore, we performed electron microscopy (EM) on sorted KCs after the HFHS diet. WT-KCs from steatotic livers had few lipid droplets despite the increased lipid content in the liver (Figure 5A). In contrast, TFEB-KCs had more lipid droplets and lipid droplet area after the HFHS diet (Figures 5A–D). Most of the lipids of the droplets in transgenic KCs were in vacoules (Figures 5A, E, F).

To assess whether TFEB changes the capacity for FA uptake or storage, we utilized BMDMs from Mac^Tfeb^ mice. The extent of *Tfeb* upregulation was comparable between the BMDMs from Mac^Tfeb^ mice and KCs from the KC^Tfeb^ mice (Figures 5G, 1C). BMDMs were incubated with BODIPY-labeled C16:0 mixed with unlabeled oleic acid (C18:1), and lipid accumulation was quantified. TFEB-BMDMs had significantly more fluorescent lipid at 24 hours, and this continued out to 54 hours, a timepoint where the signal in the WT BMDM had begun to plateau (Figures 5H, I). TFEB-BMDMs also had greater mitochondrial oxidative capacity (Figure 5J and Supplemental Figure S5F, http://links.lww.com/HC9/C188). Thus, TFEB enhances the capacity for lipid storage and FAO in macrophages.

Lysosomal acid lipase (LAL) breaks down lipid droplets in the lysosome, and liberated FAs can be delivered to the mitochondria via the carnitine palmitoyltransferase (CPT) system for oxidation (Figure 5K). To evaluate the role of lysosomal lipolysis and/or mitochondrial oxidation in KCs for the attenuated hepatic steatosis phenotype, we generated and validated mice in which TFEB overexpression was combined with KC-specific *Cpt2* knockout (KO; KC^Tfeb^CPT2^fl/fl^) to disrupt FAO or KC-specific *Lipa* KO (KC^Tfeb^LAL^fl/fl^) to prevent lysosomal lipolysis (Supplemental Figure S5G, http://links.lww.com/HC9/C188). The protective effect of TFEB-KCs on hepatic steatosis was abolished in the absence of CPT2 or LAL, arguing that lipid breakdown and oxidation by TFEB-KCs reduce steatosis (Figures 5L, M and Supplemental Figures S5H–K, http://links.lww.com/HC9/C188).

Overexpression of TFEB in macrophages has been reported to protect against obesity and insulin resistance via upregulation of GDF15^25^ (Figure 5K). While *Gdf15* mRNA was upregulated in TFEB-KCs (Figures 1D, 4D), GDF15 was not elevated in the plasma of KC^Tfeb^ mice as it was in Mac^Tfeb^ mice (Figure 5N and Supplemental Figure S5L, http://links.lww.com/HC9/C188). We deleted GDF15 in TFEB-TG animals (KC^Tfeb^GDF15^fl/fl^) (Supplemental Figure S5M, http://links.lww.com/HC9/C188), and after a HFHS diet, weight gain and hepatic steatosis were similar, indicating GDF15 also contributes to the reduction in hepatic steatosis with TFEB (Supplemental Figures S5N–P, http://links.lww.com/HC9/C188).

### TFEB protects macrophages from cell death independent of lysosomal lipid metabolism

**FIGURE 6:**
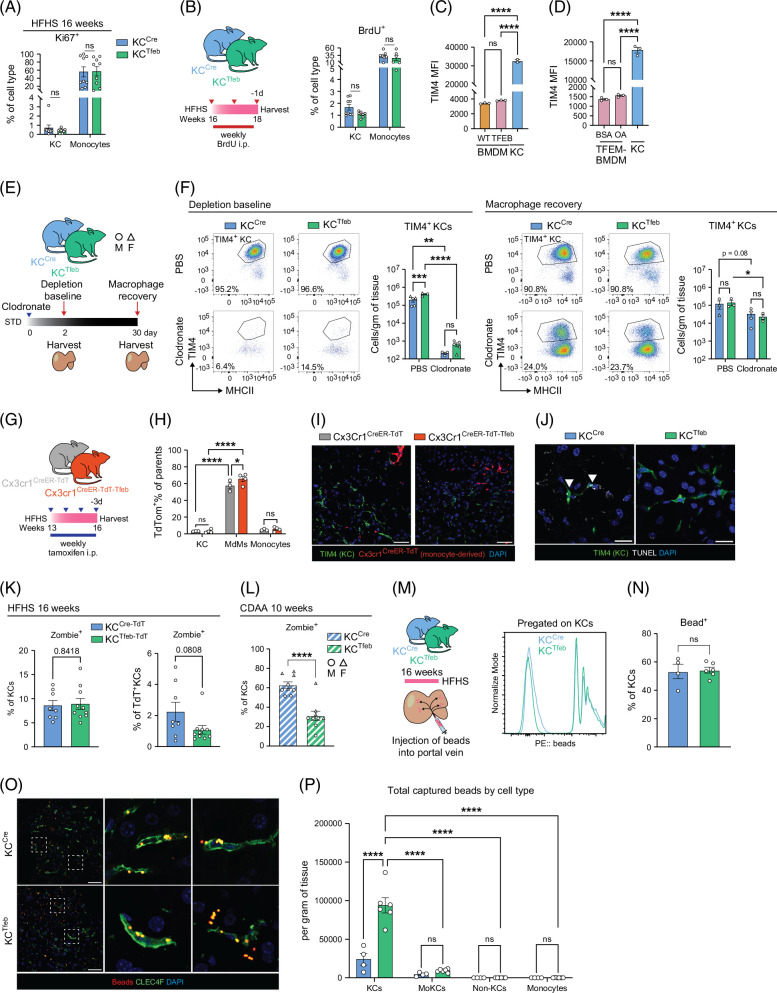
TFEB mitigates macrophage death and improves portal filtration. (A) Quantification of Ki67^+^ percentage in KCs after 16 weeks HFHS diet feeding in male KC^Cre^ and KC^Tfeb^ mice (n=9/genotype). (B) Schematic of BrdU incorporation experiment where KC^Cre^ and KC^Tfeb^ littermates fed a 16-week HFHS diet were intraperitoneally (i.p.) injected with BrdU once a week for 3 weeks, and flow cytometric quantification of BrdU^+^ percentage in cells (n=8–9/genotype). (C, D) MFI of TIM4-BV421 staining on BMDMs isolated from (C) Mac^Cre^ or Mac^Tfeb^ mice, and (D) Mac^Tfeb^ mice loaded with 250 µM oleic acid (OA): BSA for 24 hours, compared with WT-KCs. (E, F) Macrophage depletion experiment in KC^Cre^ and KC^Tfeb^ mice. (E) Experimental design. (F) Representative flow plots (pregated on F4/80^hi^CD11b^int^), average percentage of TIM4^+^ KCs as a percentage of F4/80^hi^CD11b^int^ cells, and quantification of TIM4^+^ KCs by flow cytometry. (G–I) Cx3cr1^CreER-TdT^ and Cx3cr1^CreER-TdT-Tfeb^ littermates fed HFHS diet and i.p. injected with tamoxifen once a week for 4 weeks before harvest at 16 weeks (n=3–4/group). (G) Schematic of injection regimen, (H) flow cytometric quantification of TdT^+^ percentage in cells, and (I) representative immunofluorescence images of livers. Green: TIM4; red: TdT reporter; blue: DAPI. Scale bar=50 µm. (J) Representative images of TUNEL staining identified in the livers of 16-week HFHS diet-fed KC^Cre^ and KC^Tfeb^ mice. Green: TIM4; white: TUNEL; blue: DAPI. Scale bar=20 µm. (K) Male KC^Cre-TdT^ and KC^Tfeb-TdT^ mice were placed on HFHS diet for 16 weeks, and cell death was assessed by Zombie Aqua positivity in total KCs or TdT^+^ KCs (n=8–10/genotype). (L) KC^Cre^ and KC^Tfeb^ mice were placed on a CDAA diet for 10 weeks, and cell death was assessed by Zombie Aqua positivity in total KCs (n=9/genotype). Circles represent males; triangles represent females. (M–P) KC^Cre^ and KC^Tfeb^ male mice were fed HFHS for 16 weeks, and livers were in situ injected with fluorescent beads (n=4–5/group). (M) Schematic of experiment and representative flow histogram of bead signal in KCs. (N) Percentage of KCs with single or multiple bead-positive signals. (O) Representative immunofluorescence images of bead capturing in KCs. Red: beads; green: CLEC4F; blue: DAPI. Scale bar=50 µm. (P) Total beads captured by liver myeloid cells and monocytes per gram of tissue. Values were determined by multiplying cells of interest per gram of tissue by the bead-positive percentage. Data represent individual biological replicates and are presented as means±SEM. *p-*values were calculated using (A, B, K, L, N) unpaired 2-tailed Student *t* tests, (C, D) one-way ANOVA followed by multiple *t* tests, and (F, H, P) 2-way ANOVA followed by multiple *t* tests. NS=not significant, *p<0.05, **p<0.01, ***p<0.001, and ****p<0.0001. Abbreviations: BMDMs, bone marrow-derived macrophages; BSA, bovine serum albumin; CDAA, choline-deficient amino acid-defined; KCs, Kupffer cells; HFHS, high-fat, high-sucrose, and high-cholesterol; MFI, mean fluorescent intensity; TFEB, transcription factor EB; WT, wild type.

The preservation of KCs in KC^Tfeb^ mice during MASLD could reflect increased proliferation, accelerated differentiation of MdMs to TIM4^hi^ KC, or reduced cell death. To assess proliferation, we performed Ki67 staining on KCs and observed no differences between genotypes (Figure 6A). We also injected KC^Cre^ and KC^Tfeb^ mice with BrdU after the HFHS diet and observed a slight decrease in BrdU incorporation in TFEB-KCs (Figure 6B). Thus, TFEB does not increase KC proliferation during MASH.

To determine whether TFEB accelerated the differentiation of MoKCs to TIM4^hi^ KCs, we took several approaches. First, we found that overexpression of TFEB alone or combined with exogenous lipids in BMDMs did not increase expression of the resident KC marker TIM4 (Figures 6C, D). Second, we depleted macrophages in KC^Cre^ and KC^Tfeb^ mice with clodronate liposome and assessed TIM4^hi^ KC recovery after 4 weeks (Figure 6E). Clodronate effectively depleted KCs in both genotypes, and after 4 weeks, the number of TIM4^hi^ KCs was similar between the genotypes (Figure 6F and Supplemental Figure S6A, http://links.lww.com/HC9/C188). We obtained similar results with macrophage depletion after HFHS feeding (Supplemental Figure S6B, http://links.lww.com/HC9/C188). Third, we crossed TFEB transgenic mice to animals expressing the tamoxifen-inducible Cx3Cr1–CreER system in addition to the TdT reporter (Cx3Cr1^CreER-TdT-Tfeb^). TIM4^hi^ KCs lack expression of *Cx3Cr1*; thus, this system allows for fate mapping of MdMs. Cx3Cr1^CreER-TdT-Tfeb^ and CreER-only littermates were fed an HFHS diet for 12 weeks, after which they were injected with tamoxifen once a week for 4 weeks to induce TFEB overexpression in MdMs (Figure 6G). We observed robust expression of TdT within MdMs, but the reporter was not detected in TIM4^hi^ KCs (Figures 6H, I and Supplemental Figure S6C, http://links.lww.com/HC9/C188). Together, these data indicate that TFEB overexpression does not promote rapid maturation of MdMs into TIM4^hi^ KCs.

To assess the impact of TFEB on macrophage cell death in vivo, we performed TUNEL staining in conjunction with TIM4 staining and observed fewer dying KCs in TG mice (Figure 6J). To quantify cell death, we performed Zombie staining via flow cytometry. Although total death in KCs was similar between the genotypes, cell death was specifically reduced in TdT^+^KCs, that is, cells with TFEB induction (Figure 6K). We saw similar findings with the CDAA diet (Figure 6L). Together, these data argue that TFEB preserves KC number in MASLD by reducing macrophage cell death.

TFEB deletion in KCs does not affect the survival of these cells, potentially related to compensation by other MiT transcription factor family members such as TFE3 (Supplemental Figures S6D–G, http://links.lww.com/HC9/C188).

### KC protection by TFEB improves liver filtration in MASLD

To evaluate the impact of KC preservation by TFEB on liver filtration, we established an *in situ* assay in which fluorescent beads were injected into the portal vein of animals fed an STD or HFHS diet (Supplemental Figure S6K, http://links.lww.com/HC9/C188). Using flow cytometry, we quantified bead capture (Supplemental Figure S6L, http://links.lww.com/HC9/C188). After HFHS feeding, KCs were less likely to take up single or multiple bead(s) (Supplemental Figures S6M, N, http://links.lww.com/HC9/C188), yet KCs were the most proficient at capturing beads as compared with MdMs and monocytes (Supplemental Figures S6O, P, http://links.lww.com/HC9/C188).

We then repeated this phagocytosis assay with KC^Cre^ and KC^Tfeb^ mice after the HFHS diet (Figure 6M). TFEB overexpression preserved KC numbers (Supplemental Figure S6Q, http://links.lww.com/HC9/C188) but did not alter their phagocytic capacity (Figures 6M–O). However, the total number of beads captured in the liver was significantly increased in KC^Tfeb^ mice (Figure 6P). Thus, enhancing KC survival in the KC^Tfeb^ mice facilitates the uptake of particulate from liver sinusoids. As before, we observed that KCs are more efficient at ingesting beads than MoKCs and non-KCs (Figure 6P), suggesting that KC filtration cannot be fully replaced by other macrophage populations.

### TFEB induction reduces oxidative stress-mediated cell death in macrophages

**FIGURE 7:**
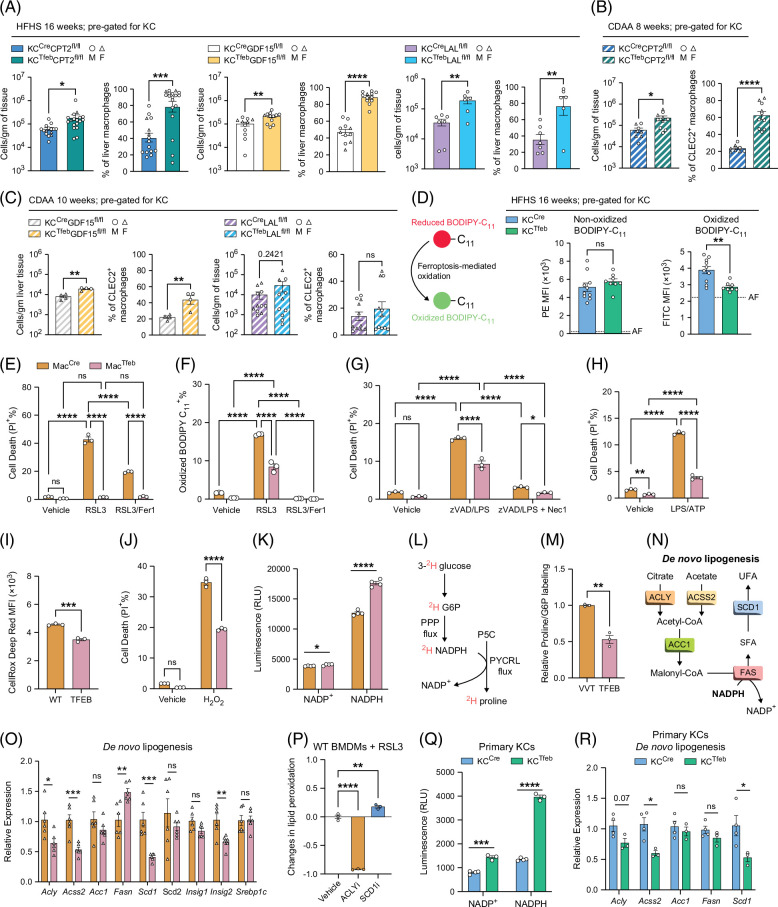
TFEB-mediated protection of macrophages in vivo and in vitro. (A–C) Flow cytometric quantification of KCs per gram of liver and percentages isolated from various mouse lines fed. (A) HFHS diet for 16 weeks (n=6–18/group) or (B, C) CDAA for 8–10 weeks (n=4–12/group); circles represent males, and the triangles represent females. (D) MFI of BODIPY-C_11_ staining in KCs isolated from KC^Cre^ and KC^Tfeb^ mice fed HFHS for 16 weeks (n=8–10/group). AF=autofluorescence of KCs determined from unstained samples. (E–M) In vitro experiments using BMDMs isolated from Mac^Cre^ and Mac^Tfeb^ mice. Flow cytometric percentage of (E) propidium iodide^+^ (PI^+^) signal and (F) oxidized BODIPY-C11^+^ signal in cells treated with 5 µM RSL3±5 µM Fer1 for 24 hours and 3 hours, respectively. Vehicle=DMSO. (G) Cells were stimulated with 20 µM zVAD and 100 ng/mL LPS±20 µM necrostatin-1 (Nec1, necroptosis inhibitor) for 24 hours, and cell death (necroptosis) was measured by PI. Vehicle=DMSO/PBS. (H) Cells were stimulated with 500 ng/mL LPS for 4 hours, then 5 mM ATP for 1 hour, and cell death (pyroptosis) was measured by PI. Vehicle=PBS. (I) MFI of CellRox Deep Red signal in WT-BMDMs or TFEB-BMDMs treated with RSL3±Fer1 for 3 hours. Representative histograms are shown. (J) Cells were stimulated with 2.5 mM H_2_O_2_ for 2 hours, and cell death was measured by PI. (K) NADP^+^ and NADPH levels in BMDMs. (L, M) Assessment of cytosolic NADPH flux through isotope tracing. (L) Schematic illustration of [3-^2^H] glucose incorporation into proline as a tracer strategy to assess cytosolic NADPH flux. (M) Relative ratio of proline to glucose-6-phosphate (G6P) with ^2^H labeling. (N) Schematic of de novo lipogenesis. (O) Gene expression of DNL-related enzymes in BMDMs. (P) Change in oxidized BODIPY-C_11_ signal between inhibitor-treated and vehicle-treated WT-BMDMs 3 hours post-RSL3 stimulation. Cells were pretreated with respective inhibitors for 16 hours before co-treatment with RSL3. (Q, R) Ex vivo experiments with primary KCs isolated from male KC^Cre^ and KC^Tfeb^ mice (n=3–4/genotype). (Q) NADP^+^ and NADPH levels in primary KCs. (R) Gene expression of DNL-related enzymes in primary KCs. Data represent (A–D, Q, R) individual biological replicates, (E–K, M, P) technical replicates, or (O) biological duplicates with technical triplicates, and are presented as means±SEM. *p*-values were calculated using (A–D, I, K, M, O, Q, R) unpaired 2-tailed Student *t* tests, (E–H, J) 2-way ANOVA followed by multiple *t* tests, and (P) 1-way ANOVA followed by multiple *t* tests. NS=not significant, *p<0.05, **p<0.01, ***p<0.001, and ****p<0.0001. Abbreviations: BMDM, bone marrow–derived macrophages; CDAA, choline-deficient amino acid-defined; DNL, de novo lipogenesis; HFHS, high-fat, high-sucrose, and high-cholesterol; KCs, Kupffer cells; LPS, lipopolysaccharide; MFI, mean fluorescent intensity; PI, propidium iodide; SFA, saturated fatty acid; TFEB, transcription factor EB; UFA, unsaturated fatty acid; WT, wild type.

We next investigated the mechanism through which TFEB induction attenuates macrophage cell death. First, we found TFEB still rescued KCs after deletion of CPT2, LAL, or GDF15 (Figure 7A) with HFHS diet feeding. This was also true in the CDAA diet for TFEB-KCs with CPT2 or GDF15 deletion (Figures 7B, C). MdM recruitment also followed a similar pattern (Supplemental Figures S7A–C, http://links.lww.com/HC9/C188).

Ferroptosis is a mode of cell death driven by lipid peroxidation that has recently been shown to contribute to KC death during MASLD.^20^ In ferroptosis, the oxidation of polyunsaturated fatty acids triggers loss of membrane integrity and cell death. Using BODIPY-C_11_, a lipid peroxidation sensor, we found that TFEB-KCs had decreased lipid peroxidation compared with WT-KCs (Figure 7D). To further assess this in vitro, we treated WT and TFEB-BMDMs with the ferroptosis inducer RSL3. RSL3 induced cell death in WT-BMDMs, but cell death was significantly attenuated in TFEB-BMDMs or by the ferroptosis inhibitor ferrostatin (Fer1; Figure 7E). Lipid peroxidation was also attenuated in TFEB-BMDMs and completely prevented by Fer1 (Figure 7F). A similar phenotype was observed with another inducer of ferroptosis, ML162 (Supplemental Figure S7D, http://links.lww.com/HC9/C188). Time course analysis with RSL3 demonstrated that TFEB-BMDMs, but not WT cells, suppressed lipid peroxidation and cell death over 24 hours (Supplemental Figure S7E, http://links.lww.com/HC9/C188). In addition to ferroptosis, TFEB-BMDMs were protected from necroptosis and pyroptosis (Figures 7G, H). NLRP3-mediated IL-1β release was also dampened in TFEB-BMDMs (Supplemental Figure S7F, http://links.lww.com/HC9/C188). The protection against multiple death pathways suggested that TFEB modulated a common pathway of cell death. Reactive oxygen species (ROS) is a shared among various modes of cell death.^30^ Utilizing the general ROS sensors CellRox and DCF, we found that TFEB-BMDMs have reduced levels of ROS (Figure 7I and Supplemental Figure S7G, http://links.lww.com/HC9/C188). Furthermore, TFEB-BMDMs were resistant to cell death induced by hydrogen peroxide (H_2_O_2_)-mediated oxidative stress (Figure 7J). Taken together, TFEB reduces ROS accumulation and protects macrophages from cell death.

### TFEB promotes NADPH accumulation by suppressing *de novo* lipogenesis

To investigate the mechanism of ROS-resistance in TFEB-macrophages, we assessed the expression of antioxidant genes and found that they were all decreased in TFEB-BMDMs (Supplemental Figure S8A, http://links.lww.com/HC9/C188). NADPH provides reducing equivalents to fuel antioxidant enzymes. Interestingly, NADPH levels were increased in TFEB-BMDMs compared with WT cells (Figure 7K and Supplemental Figure S8B, http://links.lww.com/HC9/C188). The pentose phosphate pathway (PPP) is a major pathway responsible for the generation of NADPH in the cytosol^31^ (Supplemental Figure S8C, http://links.lww.com/HC9/C188). However, the expression of genes involved in the PPP was reduced in TFEB-BMDMs (Supplemental Figure S8C, http://links.lww.com/HC9/C188). To assess cytosolic NADPH production, we performed an isotope tracing experiment with [3-^2^H] glucose labeling in BMDMs. This approach leverages the fact that cytosolic proline synthesis utilizes NADPH as a cofactor, thus allowing us to infer changes in cytosolic NADPH flux as previously established^32^ (Figure 7N). We uncovered reduced deuterium enrichment in proline in TFEB-BMDMs (Figure 7M and Supplemental Figure S8D, http://links.lww.com/HC9/C188), indicating reduced cytosolic NADPH production.^32^ Likewise, ribose-5-phosphate and ribulose-5-phosphate, the end products of oxidative PPP, were unchanged (Supplemental Figure S8E, http://links.lww.com/HC9/C188). Thus, TFEB does not increase NADPH production.

*De novo* lipogenesis (DNL) is a major NADPH-consuming pathway^31^ (Figure 7N). The mRNA expression of core DNL enzymes *Acly*, *Acss2*, and *Scd1* was decreased in TFEB-BMDMs (Figure 7O). ATP-citrate lyase (ACLY) is upstream of the main NADPH-consuming step of DNL, whereas stearoyl-CoA desaturase 1 (SCD1) is downstream (Figure 7N). As both *Acly* and *Scd1* are reduced in TFEB-BMDMs, we utilized inhibitors of ACLY or SCD1, followed by RSL3 to induce ferroptosis. Inhibition of ACLY with BMS303141 strongly attenuated lipid peroxidation, whereas SCD1 inhibition with CAY10566 mildly exacerbated it (Figure 7P and Supplemental Figure S7F, http://links.lww.com/HC9/C188), consistent with the idea that NADPH consumption by DNL can sensitize macrophages to ferroptosis. To confirm these findings in vivo, we quantified NADPH levels in freshly isolated primary KCs from KC^Cre^ and KC^Tfeb^ mice. We found that TFEB-overexpressing KCs had an increase in cellular NADPH (Figure 7Q). The expression of PPP-related genes (Supplemental Figure S8G, http://links.lww.com/HC9/C188) and DNL-related genes (Figure 7R) was also lower in TFEB-KCs compared with WT.

## DISCUSSION

The study of macrophages in MASLD has largely focused on recruited MdMs as drivers of tissue pathology. While metabolic liver disease has also been associated with KC loss, the local and systemic consequences of resident macrophage attrition have not been investigated. KCs are important for liver homeostasis and immune regulation, leading us to hypothesize that restoring KCs in MASLD could be an approach to attenuate liver pathology. We report here that activating TFEB in resident KCs during MASLD reprograms their metabolism, resulting in reduced liver steatosis and improved KC survival.

Our data adds to a growing body of literature supporting a beneficial role for macrophage TFEB in metabolic disease. Previous studies using LysM–Cre to drive TFEB overexpression in myeloid cells revealed protection against atherosclerosis, diet-induced obesity, and insulin resistance.^25^ In addition, it was recently shown that loss of Hif-2α in macrophages increases TFEB activation in KCs and reduces liver steatosis and damage.^19^ In obesity and insulin resistance, the protective effect of TFEB-overexpression in macrophages using a LysM–Cre system was mediated by increased release of the cytokine GDF15.^25^ While the expression of *Gdf15* mRNA was also increased in TFEB-KCs, we did not observe increased levels of GDF15 in circulation. In line with this, KC^Tfeb^ mice were not lean and had similar systemic metabolic dysfunction compared with controls. Instead, GDF15 derived from TFEB-overexpressing KCs appears to contribute to the regulation of steatosis, possibly via the ability of this cytokine to enhance macrophage FAO capacity.^33^ Taken together, these data indicate that activating TFEB in macrophages is beneficial in obesity-related disorders through GDF15-dependent and independent mechanisms.

Several recent studies have suggested that macrophages interact with parenchymal cells to orchestrate lipid handling. This is most notable in the adipose tissue, where ATMs take up and process adipocyte-derived extracellular vesicles that are rich in TAG.^34^ We recently demonstrated that MdMs also take up lipids released from hepatocytes early in MASLD.^27^ In contrast, KCs isolated from steatotic livers contain fewer lipid droplets and appear less adapted to handle excess fatty acids. We found that TFEB activation in KCs enhanced their ability to metabolize lipids from hepatocytes, and this resulted in reduced steatosis and liver injury. This conclusion is supported by the following observations: (1) KC^Tfeb^ mice had reduced macrovesicular steatosis and decreased levels of several TAG species in the liver without directly altering hepatocyte lipid metabolic gene expression. (2) RNAseq of isolated TFEB-KCs highlighted enhanced expression of genes involved in lipid uptake (*Cd36*) and metabolism (*Lipa*). (3) EM of TFEB-KCs revealed a significant increase in the number of lipid droplets in vacuoles compared with WT-KCs. (4) Disruption of FAO or lysosomal lipolysis in TFEB-KCs mitigated the protective effect of TFEB on hepatic steatosis. Thus, KCs are poorly adapted to the lipid-rich environment of MASLD but can be programmed by TFEB induction to enhance lipid handling and improve hepatic steatosis. TFEB was also found to increase KC resistance to cell death in mouse models of MASLD and MASH. Importantly, TFEB protects KCs in both obesogenic and inflammatory diet models of MASLD. Ferroptosis is a mode of cell death that occurs via iron-mediated lipid peroxidation, which has been implicated in KC death.^20^ We found that TFEB overexpression protected macrophages from not only ferroptotic cell death but also necroptosis and pyroptosis, through protection against ROS, a shared mechanism involved in these forms of cell death. This observed antioxidative activity in TFEB-overexpressing macrophages was linked to increased NADPH, which appears to accumulate due to reduced consumption by DNL. TFE3, a related family member of TFEB, has also been reported to reduce DNL in hepatocytes.^35^ Inhibiting ACLY, which blocks DNL upstream of NADPH consumption, protected WT macrophages against lipid peroxidation and ferroptosis. Thus, metabolic reprogramming via TFEB overexpression reduces DNL, which protects macrophages from oxidative stress via increasing NADPH. We subsequently validated the reduced expression of DNL genes and enhanced levels of NADPH in primary TFEB-KCs. Taken together, our data argue that TFEB induction in KCs alters lipid biosynthesis and reduces oxidative stress, thereby improving KC fitness in MASLD.

MASLD is associated with perturbations in gut integrity, which influences the contents of portal blood entering the liver.^36^ The enrichment of KCs in the periportal region serves as a firewall to capture infiltrating bacteria and prevent systemic dissemination.^7^^,^^9^^,^^37^ We found that preserving KC numbers through TFEB induction in the MASLD liver allowed them to maintain their fundamental role as a circulation filter. Our data also highlighted the fact that compared with KCs, recruited macrophages are less efficient at capturing particulate antigen. This result may reflect both tissue localization and/or differences in phagocytic capacity; however, it supports the concept that recruited macrophages are unable to immediately fulfill the responsibility of the KC network.

MASLD and its sequelae remain the leading causes of liver transplant and cardiovascular death. Our data demonstrate that harnessing lysosomal lipid metabolism through TFEB induction promotes KC fitness in MASLD, leading to improved liver steatosis. In mice fed a HFHS diet that models obesity-driven MASLD, TFEB induction in KCs consistently led to a ~68% reduction in inflammatory MdM infiltration and decreased expression of inflammatory cytokine *Il1b* during early disease. As fibrosis was minimal with this diet, we utilized a choline-deficient (CDAA) diet to induce fibrosis in a shorter duration. CDAA diet causes more hepatocyte injury than HFHS and is associated with a massive influx of immature MdMs. While TFEB-overexpressing KCs had a survival advantage in this diet, there was still some loss of the resident macrophages. However, the influx of immature MdMs and fibrosis was unchanged in KC^Tfeb^ mice. This interpretation must be made with caution, as the CDAA diet is distinct from the obesity-driven pathogenesis of human MASH. Further investigation using obesity-inducing MASH diet models for longer periods will be important to assess the relevance of our findings on MASH-fibrosis.

TFEB induction and its downstream impact on lipid metabolism and lysosome biology have considerable overlap between mouse and human cell lines and tissues.^21^^,^^25^^,^^35^^,^^38^^,^^39^ We and others have also reported that human and murine KCs share similarities in their markers and biology.^14^^,^^27^ While our findings support a beneficial role of TFEB induction in MASLD, additional studies in human macrophages under metabolic stress will be necessary to assess the translational potential of targeting TFEB in macrophages. Clinically available TFEB activators such as trehalose^40^ and mTOR antagonists^35^ may offer therapeutic opportunities. Evaluating their interaction with promising MASH therapies such as GLP-1 receptor and THR agonists will be critical. In summary, we demonstrate that TFEB protects KCs in MASLD, reduces liver steatosis, and preserves liver filtration.

## Supplementary Material
