# Supplementary material for: Induction of TFEB promotes Kupffer cell survival and reduces lipid accumulation in MASLD
Source: Hepatol Commun. 2025 Nov 24;9(12):e0853. doi: 10.1097/HC9.0000000000000853 (PMC12657046; doi:10.1097/HC9.0000000000000853)
Supplement: Supplementary file 1 [file hc9-9-e0853-s001.pdf]

HEP4-25-1160

Title: Induction of TFEB promotes Kupffer cell survival and reduces lipid accumulation in MASLD

Supplementary Materials

Supplemental Methods

Fig. S1 to S8

Supplemental References

Supplemental Table 1: Significantly altered genes from bulk RNAseq of WT and TFEB-KCs purified from STD or HFHS-fed KC^{Cre} and KC^{Tfeb} mice (Figure 4)

Supplemental Table 2: qPCR primers

Supplemental Methods

Study Design

Our study generated a mouse model in which overexpression of TFEB is specific to KCs to investigate the impact of TFEB induction on KC biology and disease outcome during MASLD. We fed mice two different MASLD/MASH-inducing diets and characterized KC function and survival using flow cytometry, imaging, and lipidomics. Male mice were predominately used for obesogenic diet studies and both female and male mice were used for fibrogenic diet studies. We also generated additional depletion of lipid metabolic machinery in the KC^{Tfeb} mice to investigate the mechanism for TFEB's influence *in vivo*. Complementary to our KC-specific induction of TFEB, BMDMs generated from pan-macrophage Cre-driven TFEB-overexpressing mice were utilized for *in vitro* studies. Sample size determination was informed by prior studies. Sample exclusion criteria (liver cancer, bite wounds, splenomegaly, and other unexpected organ abnormalities) were determined before data acquisition. Blinding was implemented whenever possible for data collection and researchers were unblinded for data analyses.

Mice

For tamoxifen treatment, 20 μg tamoxifen per gram of animal was prepared fresh in corn oil by incubation at 42°C with agitation until dissolution prior to intraperitoneal (i.p.) injection. For BrdU experiment, mice were injected i.p. with 100 mg BrdU per kg of animal. For macrophage depletion experiment, 150-200 μL of clodronate liposome (Tribio Science, F70101C-N) was injected i.p. into animals. All injections and euthanasia procedures were carried out by trained personnel to minimize animal distress. Littermates with different genotypes were co-housed to minimize microbiota difference. All mice were housed in specific pathogen-free conditions with 12-hour light/dark cycle. Mice of 8-12 weeks of age were used for diet studies.

Tissue harvest and processing

Animals were fasted for 4h before tissue harvest. Livers were collected based on our previously published protocol[41]. Briefly, animals were euthanized by CO_2 inhalation with cervical dislocation as secondary death confirmation. The liver was perfused with PBS through the portal vein and then dissected out. Gallbladder was discarded. Approximately 1g of the left lobe was weighted, minced, and transferred to collagenase- and DNaseI-containing (0.75 mg/mL and 50 μg/mL, respectively) media on ice until all the organs from all the animals were harvested. Liver in enzymatic solution was then placed on a rotating shaker for 30 min at 37°C for digestion. Liver digest was then passed through 70 μm cell strainer and washed with cold complete DMEM containing fetal bovine serum (FBS) to deactivate enzymatic activities. Hepatocytes were pelleted by centrifuging at 50 xg, 3 min, at 4°C and discarded. The supernatant was transferred to a new 50 mL falcon tube and non-parenchymal cells (NPC) were pelleted at 900 rpm, 7 min, at 4°C. After discarding supernatant from the NPC pellet, red blood cells were lysed by resuspending cell pellets with 1 mL of ACK lysis buffer (Corning) and incubated for 5 min at room temperature. Ten mL of PBS was then added, and cells were re-pelleted at 900 rpm, 7 min, at 4°C. This cell pellet was then used for downstream analyses such as flow cytometry.

Cell culture

BMDMs were generated from the femur and tibia of mice. Briefly, sterile PBS was then injected into each bone to flush out the bone marrows. Marrows were pelleted at 950 rpm, 7 min, 4°C and

resuspended in 10% CMG-conditioned complete DMEM (high glucose DMEM with 10% fetal bovine serum (FBS), 1% 5000 U/mL Penicillin-Streptomycin (P/S), 1% 200 mM L-glutamine, and 1% 100 mM sodium pyruvate) for plating on bacterial culture plates for 6 days. Additional 10% CMG-conditioned DMEM was supplemented on days 3 and 5. For experiments, cells were counted for plating in 5% CMG-conditioned DMEM on day 6. CMG14-12 cell line was expanded to confluency in complete DMEM and conditioned media were collected and filtered every other day.

Flow cytometry and sorting (FACS)

Cells were resuspended in 1:250 ZombieAqua BV510 (in PBS) and incubated on ice for 15min in the dark. After washing with FACS buffer (PBS + 2mM EDTA + 0.5% BSA w/v), cells were re-pelleted and incubated with 1:10 Fc Block (in FACS buffer) for 5min on ice in the dark. Then 90uL of antibody cocktail was added to the cells with Fc Block for 30-60min incubation on ice in the dark. For intracellular staining with anti-Ki67 (BioLegend) or anti-BrdU (BD Biosciences Cat#552598), cells were permeabilized and stained following the manufacturer's protocol. Cells were then washed with FACS buffer and resuspended in 300-500uL FACS buffer for data acquisition using a BD Fortessa X20 flow cytometer. For cell sorting, a BD FACSAriaII or BD FACSSymphony S6 was utilized to collect KCs (singlet, live, $CD45^{+}$ $F480^{hi}$ $CD11b^{int}$ $TIM4^{+}$ $VSIG4^{+}$) or recruited MdMs (singlet, live, $CD45^{+}F480^{hi}$ $CD11b^{hi/int}MHCII^{+}Ly6C^{-}CLEC2^{+/-}$ $CD11c^{-}TIM4^{-}VSIG4^{-}$). Data analyses were performed using FlowJo software.

Endolysosomal assessment, ferroptosis detection in primary cells, and in situ bead injection

Livers were perfused with PBS followed by 37°C collagenase A (1 mg/mL) until tissue disintegrated. The liver was carefully dissected out and torn into smaller pieces in the collagenase-containing media, and put on a 37°C air shaker for additional digestion for 20min. After differential centrifugation described above to remove hepatocytes and pellet NPCs, NPCs were resuspended in 5 mL of 35% Percoll and layered onto 5 mL 70% Percoll and centrifuged for 20min at 2200 rpm at room temperature without break or acceleration. The interlayer cell suspension (enriched in macrophages) is collected into a new tube, washed with 10 mL FBS-containing DMEM, and repelleted at 900 rpm for 7 min at 4°C. Cells were counted and ~4 x 10^5 cells were incubated with 250 µL of various substrates prepared in serum-containing DMEM.

DQ-OVA (5 µg/mL, ThermoFisher, Cat# D12053), pHrodo-red (50 µg/mL, ThermoFisher, Cat#P10361), and TMR Dextran-1000MV (50 µg/mL, Invitrogen, Cat#D1817) were incubated with cells for 30 min at 37°C. Lysotracker green (1:2000, Invitrogen, Cat#L7526) was incubated with cells for 5 min at 37°C. For *ex vivo* lipid uptake assay, BODIPY-C_{16} (1 µM, ThermoFisher, Cat#D3821) was incubated for 1 min at 37°C followed by quenching with 500 µM of phloretin. For ferroptosis detection in KCs, NPCs were isolated based on the methods above, and incubated with 100uL of BODIPY 581/591 C_{11} (5 µM, ThermoFisher, D3861) in FBS-containing DMEM for 30min at 37°C. Afterward, cells were washed with PBS and subjected to antibody staining for flow cytometry.

For *in situ* injection of fluorescent beads, the mouse was euthanized, and liver was first perfused with PBS. Then 5 mL of pre-warmed, 37°C fluorescent beads (ThermoFisher, Cat#13083) prepared at 10^8 beads/mL in PBS was slowly injected into the portal vein of the mouse over 1 min. Meanwhile, the inferior vena cava (IVC) was clamped to allow for saturation of the bead solution in the liver for 5 min. Then the IVC clamp was released, and the liver was perfused with PBS for 2 min to wash off unattached beads. The liver was dissected out and incubated in 5 mL of FBS-containing DMEM for 30 min at 37°C. Then parts of the livers were either fixed for immunofluorescence or digested with collagenase and DNase I following the protocol above for flow cytometry. To calculate the total beads captured per gram of tissue, beads$^+$ percentages were multiplied by respective cell numbers per gram of tissue.

Electron Microscopy

KCs were FACS-purified based on TIM4 expression as described above. For ultrastructural analyses, cells were fixed in 2% paraformaldehyde/2.5% glutaraldehyde (Ted Pella Inc., Redding, CA) in 100 mM sodium cacodylate buffer for 2h at room temperature. Samples were washed in sodium cacodylate buffer and postfixed in 1% osmium tetroxide (Ted Pella Inc.) for 1h at room temperature. Samples were then rinsed extensively in dH20 prior to en bloc staining with 1% aqueous uranyl acetate (Electron Microscopy Sciences, Hatfield, PA) for 1 h. Following several rinses in dH20, samples were dehydrated in a graded series of ethanol, and embedded in Eponate 12 resin (Ted Pella Inc). Ultrathin sections of 95 nm were cut with a Leica Ultracut UCT ultramicrotome (Leica Microsystems Inc., Bannockburn, IL), stained with uranyl acetate and lead citrate, and viewed on a JEOL 1200 EX transmission electron microscope

(JEOL USA Inc., Peabody, MA) equipped with an AMT 8 megapixel digital camera and AMT Image Capture Engine V602 software (Advanced Microscopy Techniques, Woburn, MA). For quantitation of lipid droplets, 20 to 40 cells that had a nucleus cut in cross-section (indicating cross-section through the middle of cell) were randomly chosen, and images of each cell were taken at a magnification of 5,000X. The cross-sectional area of each of the lipid droplets and the cytosol of corresponding cell were determined using Image J 1.38g (National Institutes of Health, USA, customized for AMT images). Data is expressed as total number of lipid droplets for each cell type, and the total cross-sectional area of lipid droplets per total area of cytosol for each cell type. Distribution histogram was generated using R ggplot2 package.

Bulk RNA sequencing

Total RNA integrity was determined using Agilent Bioanalyzer or 4200 Tapestation. Library preparation was performed with 10ng of total RNA with a Bioanalyzer RIN score greater than 8.0. ds-cDNA was prepared using the SMARTer Ultra Low RNA kit for Illumina Sequencing (Takara-Clontech) per manufacturer's protocol. cDNA was fragmented using a Covaris E220 sonicator using peak incident power 18, duty factor 20%, cycles per burst 50 for 120 seconds. cDNA was blunt ended, had an A base added to the 3' ends, and then had Illumina sequencing adapters ligated to the ends. Ligated fragments were then amplified for 12-15 cycles using primers incorporating unique dual index tags. Fragments were sequenced on an Illumina NovaSeq-6000 using paired end reads extending 150 bases. Basecalls and demultiplexing were performed with Illumina's bcl2fastq software and a custom python demultiplexing program with a maximum of one mismatch in the indexing read. RNA-seq reads were then aligned to the Ensembl release 76 primary assembly with STAR version 2.5.1a[42]. Gene counts were derived from the number of uniquely aligned unambiguous reads by Subread:featureCount version 1.4.6-p5[43]. Isoform expression of known Ensembl transcripts were estimated with Salmon version 0.8.2[44]. Sequencing performance was assessed for the total number of aligned reads, total number of uniquely aligned reads, and features detected. The ribosomal fraction, known junction saturation, and read distribution over known gene models were quantified with RSeQC version 2.6.2[45].

All gene counts were then imported into the R/Bioconductor package EdgeR[46] and TMM normalization size factors were calculated to adjust for samples for differences in library size.

Ribosomal genes and genes not expressed in the smallest group size minus one samples greater than one count-per-million were excluded from further analysis. The TMM size factors and the matrix of counts were then imported into the R/Bioconductor package Limma[47]. Weighted likelihoods based on the observed mean-variance relationship of every gene and sample were then calculated for all samples with the voomWithQualityWeights[48]. The performance of all genes was assessed with plots of the residual standard deviation of every gene to their average log-count with a robustly fitted trend line of the residuals. Differential expression analysis was then performed to analyze for differences between conditions and the results were filtered for only those genes with Benjamini-Hochberg false-discovery rate adjusted p-values less than or equal to 0.05.

The R EnhancedVolcano package were utilized to generate volcano plots with abs(log2FC) >0 and p-values < 0.05 as cutoff. Gene set-based analyses were performed by entering DEGs [abs(log2FC) >0 and p-values < 0.05] into the ConsensusPathDB (http://cpdb.molgen.mpg.de/MCPDB)[49] for over-representation analysis in KEGG and Reactome databases with minimal overlap in gene list = 2 and p-value cut-off as 0.01. Heatmaps were generated using Phantasus[50].

Glucose tolerance test (GTT)

Mice were fasted for 16 h before GTT. On the day of GTT, the baseline glucose level of mice was measured by glucometer with a glucose strip with tail blood. Mice were subsequently injected 2 mg glucose per gram body weight intraperitoneally (10uL 20% glucose prepared in ddH_2O, filtered). Blood glucose level was then measured 30 min, 60 min, and 120 min after initial glucose injection. Mice were re-fed after the conclusion of GTT.

Plasma ALT measurement

Animal blood was collected from the inferior vena cava into a microtainer with EDTA. Serum was collected after spinning the blood down at 5000 rpm for 10 min at 4°C and stored at -80°C until assay. ALT quantification was performed using the Teco ALT reagent set (Teco Diagnostics, A524-150) following the manufacturer's instructions. Briefly, reagents A and B were mixed 5:1 (per sample) to create a working reaction solution and incubated for 10 min at 37°C. Reaction solution (100µL) was then added to 5 µL of animal serum (in duplicates) and absorbance at 340 nm was

read immediately with a TECAN Infinite 200 pro plate reader at 37°C once per minute for 10 min. The average absorbance per minute was determined and multiplied by a factor of 1768 for results in U/L.

Triglyceride and cholesterol quantification

Plasma and tissue used for triglyceride or cholesterol quantification originated from animals fasted for 4 h before tissue harvest. Tissue was homogenized in cold PBS such that the final homogenate solution contains 100mg tissue/mL. Homogenate solution was diluted 10-times for triglyceride measurement. Following dilution, lipid was solubilized with 1% sodium deoxycholate at 37°C for 5 min. Lipid was measured using Infinity Triglyceride Reagent or Cholesterol Reagent (ThermoFisher Cat# TR22421, TR13421) according to the manufacturer's protocol.

Lipidomic

Frozen liver samples (100 to 300mg) were homogenized in water (1:4 w/v) using Omni bead ruptor. Modified Bligh-Dyer method was performed to extract triglyceride (TAG) from 50 µL of homogenate after the addition of TAG (17:1/17:1/17:1) as internal standard. Quality control (QC) samples were prepared by pooling the aliquots of the study samples and were used to monitor the instrument stability. The QC was injected six times in the beginning to stabilize the instrument, and was injected between every 5 study samples. Only the lipid species with CV < 15% in QC sample were reported. The relative quantification of lipids was provided, and the data were reported as the peak area ratios of the analytes to the internal standard. Measurement of TAG was performed with a Shimadzu 10A HPLC system and a Shimadzu SIL- 20AC HT auto-sampler coupled to a 4000QTRAP mass spectrometer operated in positive multiple reaction monitoring mode. Data processing was conducted with Analyst 1.6.3.

Immunofluorescence

Right lateral lobes were fixed in 10% formalin overnight, washed, then submerged in 30% sucrose (in PBS) and stored at 4°C. For cryo-sectioning, liver lobes were washed in PBS and embedded in OCT compound. Eight µm-thick sections were cut using a cryostat (Leica Biosystems; Wetzlar, Germany) and stored at -80°C until staining. Immunofluorescence staining was carried out as previously published[41]. Briefly, sections were air-dried for 12 min, rehydrated in PBS for 5min,

and then blocked in fresh blocking buffer (PBS + Triton X-100 + BSA) for 1h at room temperature. Sections were circled by hydrophobic pen, and primary antibodies prepared in blocking buffer were then deposited onto the section for overnight incubation at 4°C. Next day, sections were washed in PBS 3 times, 5 min each, and secondary antibodies were deposited and incubated for 1 h at room temperature, in the dark. Sections were washed in PBS 3 times, 5 min each. Nuclei were stained with fresh Hoechst dye at 1:25,000 for 5 min at room temperature in the dark. Sections were then mounted with prolong gold antifade reagent. Click-iT Plus TUNEL assay kit (AF647) was utilized to detect dying KCs. Confocal images were acquired using an LSM 700 or 900 laser scanning confocal microscope (ZEISS; Jena, Germany) with 10x 0.48 N.A, 20x 0.8 N.A. or 63x 1.4 oil DIC objective at ambient temperature. Samples within an experiment were stained and acquired using the same laser intensity and gain (within the same magnification) on the same day with 4x averaging. Image brightness was uniformly optimized in Fiji (ImageJ, National Institutes of Health, Bethesda, MD, USA).

qPCR analysis

For sorted KCs, RNA was purified using the QIAGEN micro RNA kit. For other cell types, Invitrogen mini kits were used. For liver tissue, QIAGEN fibrotic tissue RNA kit was utilized to extract RNA from ~30 mg of frozen liver tissue. For cell culture, QIAGEN mini RNA kit was used. RNA concentration was determined by nanodrop spectrophotometer. Reverse transcription was performed using the High-Capacity cDNA Reverse Transcription Kit (Applied Biosystems). qPCR was performed using Power SYBR green reagent (Applied Biosystem) with a Quant Studio 3 platform. Relative gene expression was calculated using delta-delta CT methods and normalized to *36b4* expression. qPCR primers used is listed in Supplemental Table 2.

Histology

Right lobes were fixed in 10% formalin at 4°C overnight and then transferred into 70% ethanol for long-term storage at 4°C. Paraffin-embedding, sectioning, H&E, and picrosirius red staining (PSR) were performed by the Advanced Imaging and Tissue Analysis Core of the Digestive Disease Research Core Center at Washington University in St. Louis. Steatosis scores were quantified by a blinded liver pathologist using previously described criteria[51]. Macrovesicular and microvesicular steatosis were scored from 0 to 3 and quantified as percentages (<5%, grade 0; 5-

33%, grade 1; 34-66%, grade 2; >66% grade 3). Picrosirius red area was quantified blinded with ImageJ using manual thresholding on 7 images at high-power magnification per sample (randomly chosen area avoiding major vessels). Images were taken on an AxioImager M.2.

Plasma ELISA

Plasma GDF15 was measured using the mouse GDF15 DuoSet ELISA kit (R&D systems, Cat# DY6385) following manufacturer's instruction.

Seahorse cell mitostress test

BMDMs were seeded at 5 x 10^5 cells/mL onto Agilent Seahorse XF96 cell culture microplate for 3h to allow adhesion. The Mitostress test was performed according to the manufacturer's protocol for Seahorse XF Cell Mito Stress Test. Briefly, 1.3 μM Oligomycin (Cayman chemical; Cat #11341), 1μM FCCP (Sigma; Cat #C2920), and 1 μM rotenone (Sigma; Cat #8875) together with 10 μM antimycin-A (Sigma A-8674) were added to BMDMs every 18 minutes. Oxygen consumption rate (OCR) and extracellular acidification rate (ECAR) were recorded.

In vitro lipid accumulation assay

For assay with free fatty acid (FA), BMDMs were plated on non-tissue culture-treated plates or on #1.5 coverslips overnight, then incubated with 250 μM oleic acid conjugated with BSA (2:1 molar ratio) and 1 μM of BODIPY-C_{16} in 5% CMG-containing DMEM. After incubation for indicated time points, cells were washed with PBS, trypsinized, and resuspended with 1:3000 DAPI solution for flow cytometry. For imaging, cells were fixed with 4% PFA for 15 min at room temperature in the dark, followed by thorough washing with PBS. Nuclei were stained with 1:25000 Hoechst dye for 5 min at room temperature in the dark. Coverslips were then mounted to slides with prolong gold antifade reagent and stored in dark.

Cell death and ROS measurement in cell culture

BMDMs were plated on suspension plates and subsequently treated with various cell death inducers in complete DMEM without additional CMG-conditioned media. RSL3 (5 μM, Selleckchem, Cat#S8155) or ML162 (5 μM, Cayman Chemical, Cat#20455) and ferrostatin-1 (5 μM, Cayman Chemical, Cat#17729) were used to induce or inhibit ferroptosis, respectively.

zVAD(OMe)-FMK (20 µM, Santa Cruz, Cat#SC-311561) + LPS (100 ng/mL, in PBS) and necrostatin-1 (20 µM, EMD-Calbiochem, Cat#4311-88-0) were used to induce or inhibit necroptosis, respectively. DMSO was used as vehicle control. For H_2O_2 experiments, cells were treated with 2.5mM H_2O_2 for 2h; water was used as vehicle control. To inhibit ACLY and SCD1, cells were pretreated with BMS303141 (ACLYi) (40 µM) or CAY10566 (SCD1i) (10 nM) respectively for 16h, and then co-treated with RSL3 (5 µM) for 3h followed by lipid peroxidation measurement. For lipid peroxidation measurement, BODIPY 581/591 C_{11} (Invitrogen, Cat# D3861) were incubated with cells at a final concentration of 5 µM for 30min at 37°C; for cellular oxidative stress measurement, CellRox Deep Red (1 µM in serum-free DMEM) or DCF (10µM in HBSS) was incubated with cells for 30min at 37°C. Cell death was measured by adding propidium iodide solution (1:500 in FACS buffer) to cells after indicated time points. Data were acquired with a BD Canto II cytometer.

$NADP^+$ and NADPH measurement

$NADP^+$ and NADPH in BMDMs and KCs were measured by using Promega NADP/NADPH-Glo™ Assays (G9081), following manufacturer's instruction. Briefly, cells were suspended in 80µL PBS per well and lysed with equal volume of base solution containing 1% DTAB. Lysate from each sample (50 µL) was transferred into empty PCR tubes containing either nothing (base-treated samples) or 25 µL 0.4N HCl (acid-treated samples) and heated at 60C on a PCR block. After heating, samples were equilibrated to room temperature for 10 min. To the acid-treated samples, 25 µL 0.5M Trizma base was added; to base-treated samples, 50 µL 0.2N HCl/0.25M Trizma solution was added. To a 96-well white-wall assay plate, 50 µL of acid- or base-treated samples were transferred, and equal volume of $NADP^+$/NADPH-glo detection reagent was added and incubated for 30 min at room temperature. Luminescence was detected and recorded by a TECAN Infinite 200 pro plate reader. For BMDM assays, $7x10^5$ cells/mL cells in quadruplicates plated on 96-well plate was used. For KC assays, primary murine KCs were isolated as described in "Cell culture" section, with the additional step of normalizing the number of $CD45^+$ cells to $1x10^6$ cells/mL for plating onto 96-well plates in triplicates. Two hours after plating, non-adherent cells were removed and adherent cells (KCs) were lysed following assay instruction.

Metabolite measurement and isotope tracing in cells

For isotope tracing experiment, 10^6 BMDMs were cultured with 25mM of [3-^{2}H] glucose (Cambridge isotope laboratory, DLM-3557) in a labeling media consists of glucose-free DMEM (Gibco, A1443001), 10% fetal bovine serum, 5% CMG-conditioned media, 1% Penicillin-Streptomycin 5000 U/mL, 1% 200 mM L-glutamine, and 1% 100 mM sodium pyruvate, for 24 h. BMDMs were washed twice with PBS, twice with LC-MS grade water, and then quenched and collected with ice-cold LC-MS grade methanol into Eppendorf tubes. Cells were dried in a SpeedVac for 2 h and then reconstituted in 1 mL of cold methanol:acetonitrile:water in 2:2:1 ratio. Cell suspension was then vortexed, frozen in liquid nitrogen, and bath sonicated for 10 min at 25°C, for a total of 3 times. Samples were then stored at -20°C for 1 h, and centrifuged at 14,000 rpm at 4°C for 10 min. Supernatants were transferred to new Eppendorf tubes and dried by SpeedVac for 2 h, while pellets were resuspended in 200 µL of 50 mM sodium hydroxide for protein quantification using BCA assay (ThermoFisher). To the dried residues, 1 µL of water:acetonitrile in 1:2 ratio was added for every 2.5 µg of protein. The samples were then bath sonicated for 5 min at 25°C and vortexed, for a total of 2 times, and stored at 4°C for 1 h. Then samples were centrifuged at 14000 rpm at 4°C for 10 min. Pellets were discarded and supernatants were transferred into LC vials and stored at -80°C until mass spectrometry analyses.

Ultra-high-performance LC (UHPLC)/MS was performed with a ThermoScientific Vanquish Flex UHPLC system interfaced with a ThermoScientific Orbitrap ID-X Tribrid Mass Spectrometer. Hydrophilic interaction liquid chromatography (HILIC) separation was accomplished by using a HILICON iHILIC-(P) Classic column (Tvistevagen) with the following specifications: 100 × 2.1 mm, 5 µm. Mobile-phase solvents were composed of A = 20 mM ammonium bicarbonate, 0.1% ammonium hydroxide and 2.5 µM medronic acid in water:acetonitrile (95:5) and B = in acetonitrile:water (95:5). The column compartment was maintained at 45 °C for all experiments. The following linear gradient was applied at a flow rate of 250 µL min^{-1}: 0 to 1 minute: 90% B, 1 to 12 minutes: 90% to 35% B, 12 to 12.5 minutes: 35% to 25% B, 12.5 to 14.5 minutes: 25% B. The column was re-equilibrated with 20-column volumes of 90% B. The injection volume was 4 µL for all experiments. Data were collected with the following settings: spray voltage, 3.5 kV/−2.8 kV; sheath gas, 50; auxiliary gas, 10; sweep gas, 1; ion transfer tube temperature, 300 °C; vaporizer temperature, 200 °C; mass range, 67 to 1,000 Da, resolution, 120,000 (MS1), 30,000 (MS/MS); polarity, positive and negative; maximum injection time, 100 ms; isolation window, 1.6 Da. LC/MS data were processed and analyzed with the open-source Skyline software.

Statistics

All statistical analyses except for RNAseq data were performed using Prism 10.3 software. Two-tailed, unpaired t-tests were used when 2 independent groups were being compared. One-way ANOVA followed by Šídák's multiple comparison tests were utilized when more than 2 independent groups were being compared with each other or with a control group. Two-way ANOVA followed by multiple t-tests was performed whenever appropriate. Specific statistical tests and biological replicates (n) were described in the figure legends. P-value ≤ 0.05 was considered statistically significant for all analyses.

Antibodies used for flow cytometry

Target	Conjugate	Clone	Suppliers	Catalog #	Dilution
CD45	BUV395	30-F11	BD Biosciences	564279	1:100
CD45	PerCP-Cy5.5	30-F11	BioLegend	103132	1:100
CD11b	APC-Cy7	M1/70	BioLegend	101226	1:100
CD36	APC	CRF D-2712	BD Biosciences	562744	1:100
CLEC2	APC	17D9/CLEC-2	BioLegend	146105	1:100
CLEC2	PE	17D9/CLEC-2	Biolegend	146103	1:100
F4/80	APC	BM8	BioLegend	123122	1:100
F4/80	FITC	BM8	BioLegend	123107	1:100
F4/80	BV605	BM8	BioLegend	123133	1:100
TIM4	BV421	21H12	BD Biosciences	742773	1:500
TIM4	PE-Cy7	RMT4-54	BioLegend	130010	1:100
VSIG4	PE-Cy7	NLA14	Invitrogen	25-5752-80	1:200
VSIG4	FITC	NLA14	Invitrogen	53-5752-82	1:200
MHCII	BV605	M5/114.15.2	BioLegend	107639	1:300
Ly6C	BV711	HK1.4	BioLegend	128037	1:100
Ki67	BV605	16A8	BioLegend	652413	1:100

Antibodies used for immunofluorescence

Species	Target Species	Antigen	Clone	Suppliers	Cat. #	Dilution
Rat	Mouse	F4/80-Biotin	BM8	eBioscience	13-4801-85	1:200
Rat	Mouse	CD68	FA-11	Invitrogen	14-0681-82	1:200
Rat	Mouse	TIM4	RMT4-54	BioLegend	130002	1:100
Goat	Mouse	CLEC4F/CLECSF13	Polyclonal	R&D Systems	AF2784	1:100
Rabbit	Mouse	LYVE1	Polyclonal	Abcam	Ab14917	1:100
Rabbit	Mouse	TREM2	EPR23177-106	Abcam	Ab245227	1:100

Host Species	Target Species	Target	Conjugate	Suppliers	Cat. #	Dilution
Donkey	Rat	IgG(H+L)	AF 488	Invitrogen	A21208	1:500
Donkey	Goat	IgG (H+L)	AF 647	Jackson ImmunoResearch		1:500
Donkey	Rabbit	IgG (H+L)	AF594	Jackson ImmunoResearch	711-585-152	1:200
Donkey	Rabbit	IgG (H+L)	AF647	Jackson ImmunoResearch	711-605-152	1:500

Supplemental Figure 1. Related to Main Figure 1.

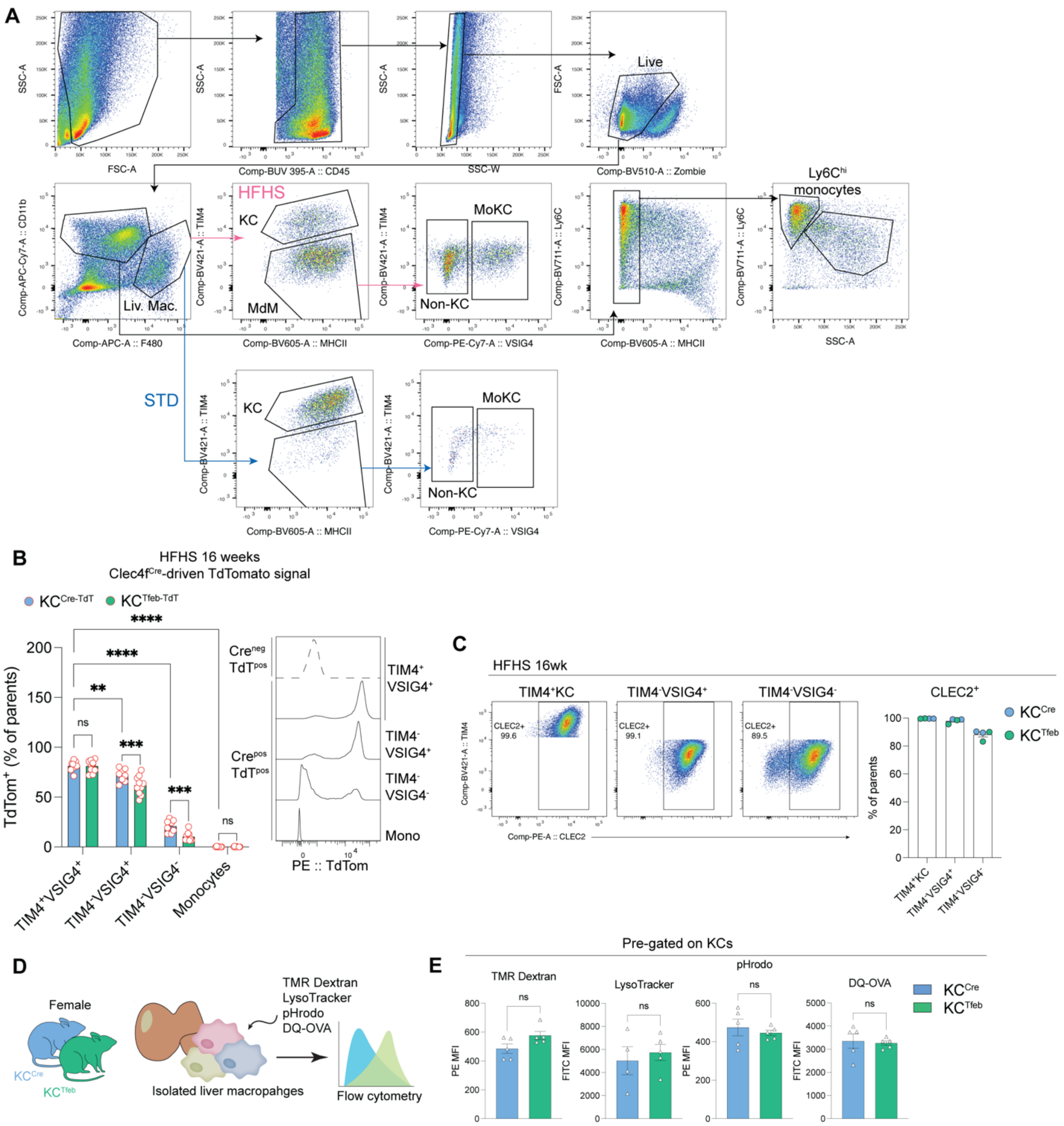

Supplemental Figure 1. Gating strategy for hepatic myeloid cells, Clec4f^{Cre} (KC^{Cre}) validation, and baseline functional assessment of TFEB-KCs. (**A**) Gating strategy for identifying liver myeloid cells and monocytes in HFHS and STD-fed mice. (**B**) TdTomato reporter signal and representative histogram in various liver myeloid cells in KC$^{Cre\text{-}TdT}$, KC$^{Tfeb\text{-}TdT}$, and no Cre control (Cre^{neg}TdT^{pos}) mice fed 16-week HFHS diet (n = 8-10/group). (**C**) Representative flow plots and quantification of CLEC2 in macrophage subsets found in KC^{Cre}

and KC^{Tfeb} mice fed 16-week HFHS diet (n = 2/group). (**D-E**) KCs were isolated from female KC^{Cre} and KC^{Tfeb} mice and incubated with various substrates to measure macrophage function and lysosomal activity (n = 5/group). (D) Schematic of experiments. (E) MFI of TMR dextran, lysotracker green, pHrodo, and DQ-OVA in WT and TFEB-KCs. Data represent individual biological replicates and are presented as means ±SEM. P-values were calculated using (B) two-way ANOVA followed by multiple t-tests, and (E) unpaired two-tailed Student's t-tests. NS = not significant, $^{*}p < 0.05$, $^{**}p < 0.01$, $^{***}p < 0.001$, $^{****}p < 0.0001$.

Supplemental Figure 2. Related to Main Figure 2.

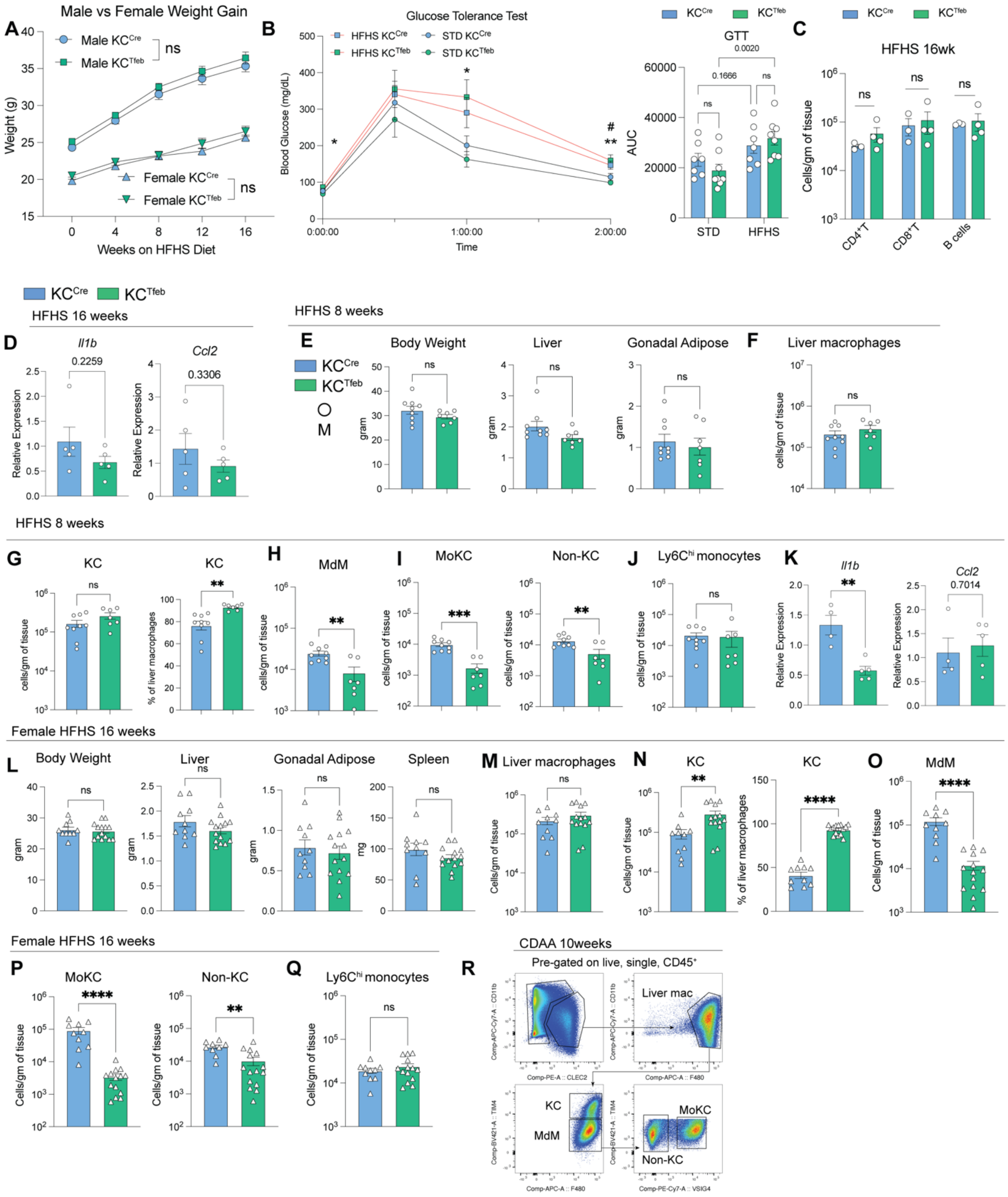

Supplemental Figure 2. Systemic obesity of male and female KC^{Cre} and KC^{Tfeb} mice fed MASLD/MASH-inducing diets. (**A**) Kinetics of weight gain for male and female KC^{Cre} and KC^{Tfeb} mice during HFHS diet feeding (n = 6-8 for females, and 26-29 for males). (**B**) Glucose

tolerance test (GTT) and quantified area under the curve (AUC) for male KC^{Cre} and KC^{Tfeb} mice fed 16 weeks HFHS diet (n = 7-8/group). # represents comparisons between STD- and HFHS-fed KC^{Tfeb} mice and * represents comparisons between STD- and HFHS-fed KC^{Cre} mice. (**D**) qPCR analysis of hepatic inflammation markers in male KC^{Cre} and KC^{Tfeb} mice fed 16 weeks HFHS diet (n = 5/group). (**C**) Flow cytometric quantification of adaptive immune cells ($CD3^+CD4^+$T cells, $CD3^+CD8^+$T cells, and $CD3^-B220^+$ B cells), in KC^{Cre} and KC^{Tfeb} mice fed 16-week HFHS diet. (**E-K**) KC^{Cre} and KC^{Tfeb} male mice were fed 8 weeks of HFHS diet (n = 7-9/group). (E) Final body and organ weights of mice. (F) Flow cytometric quantification of liver macrophages per gram of tissue. (G) KC number per gram of tissue and as a percentage of liver macrophages. (H) MdM number, (I) MoKC, non-KCs and (J) $Ly6C^{hi}$ monocytes per gram of tissue. (K) qPCR analysis of hepatic inflammation markers. (**L-Q**) Female KC^{Cre} and KC^{Tfeb} mice were fed HFHS diet for 16 weeks (n = 6-8/group). (L) Final body and organ weights of mice. (M) Flow cytometric quantification of liver macrophages per gram of tissue. (N) KC number per gram of tissue and as a percentage of liver macrophages. (O) MdM number, (P) MoKC, non-KCs, and (Q) $Ly6C^{hi}$ monocytes per gram of tissue. (**R**) Gating strategy for myeloid cells found in livers of mice fed CDAA diet utilizing CLEC2. Data represents (A, B) means ±SEM or (B-Q) individual biological replicates presented as means ±SEM. P-values were calculated using (A) unpaired two-tailed Student's t-tests between each time point, (B) two-way ANOVA followed by multiple t-tests, and (C-Q) unpaired two-tailed Student's t-tests. NS = not significant, *$p < 0.05$, **$p < 0.01$, ***$p < 0.001$, ****$p < 0.0001$.

Supplemental Figure 3. Related to main figure 3.

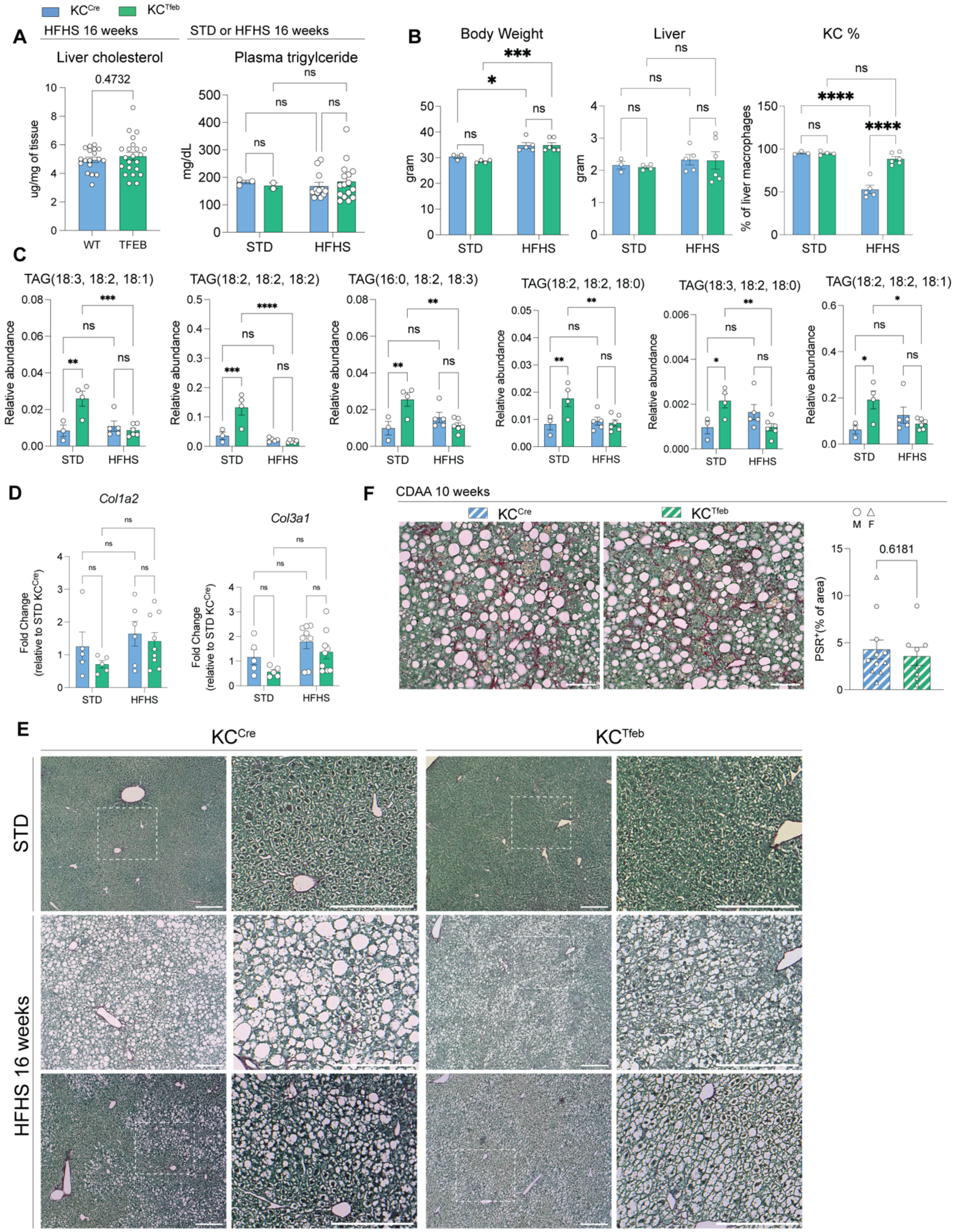

Supplemental Figure 3. Characterization of steatosis and fibrosis in KC^{Cre} and KC^{Tfeb} mice.

(**A**) Quantification of hepatic cholesterol (n = 18-22/group) and serum triglyceride (n = 2-

16/group) in KC^{Cre} and KC^{Tfeb} male mice fed 16 weeks of STD or HFHS diet (n = 18-22/group). (**B-D**) KC^{Cre} and KC^{Tfeb} male mice fed 16 weeks of STD or HFHS diet and livers were used for lipidomic. (B) Body parameters and KC preservation of mice whose livers were subjected to targeted lipidomic (n = 3-6/group). (C) TAG species with significant changes between KC^{Cre} and KC^{Tfeb} mice measured by lipidomic. (D) qPCR gene expression analyses of pathogenic collagens in whole liver tissues (n = 5-9/group). (**E**) Representative picrosirius red (PSR) staining of livers from STD or HFHS diet-fed KC^{Cre} and KC^{Tfeb} mice. Scalebar = 200µm. (**F**) Representative PSR staining of livers from CDAA diet-fed KC^{Cre} and KC^{Tfeb} mice and quantification of PSR^{+} % by averaging signal from 7 images per mouse (n = 8-11/group). Scalebar = 100µm. Data represent individual biological replicates presented as means ±SEM. P-values were calculated using (A, F) unpaired two-tailed Student's t-tests and (B-D) two-way ANOVA followed by multiple t-tests. NS = not significant, *$p < 0.05$, **$p < 0.01$, ***$p < 0.001$, ****$p < 0.0001$.

Supplemental Figure 4. Related to main figure 4.

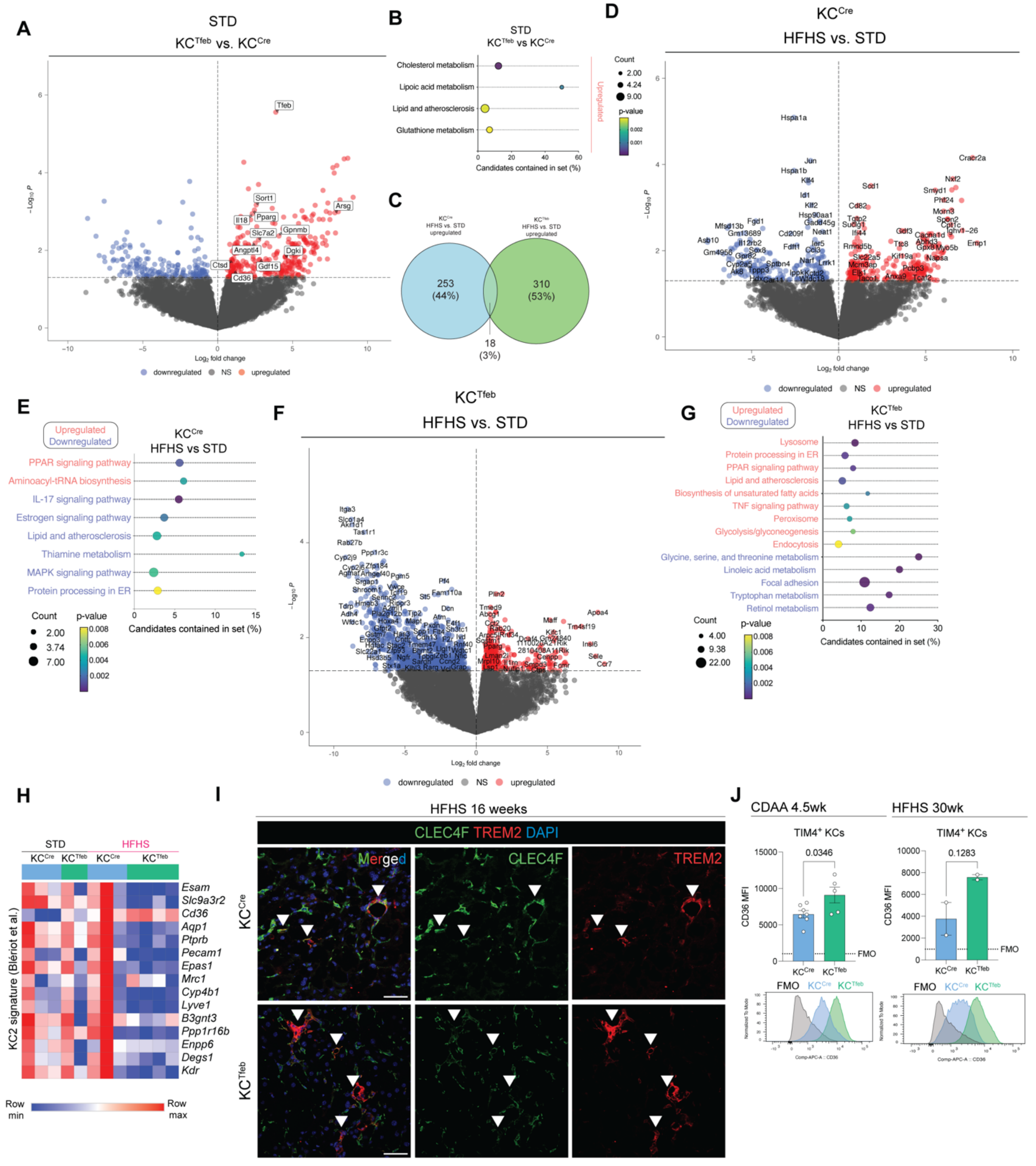

Supplemental Figure 4. Transcriptomic pathways altered by TFEB and HFHS diet in KCs. (**A-B**) DEGs in TFEB-KCs under homeostatic condition. (A) Volcano plot showing DEGs and (B) upregulated KEGG pathways in TFEB-KCs. (**C**) Venn diagram of upregulated DEGs between WT and TFEB-KCs induced by HFHS. (**D-E**) DEGs in WT-KCs after HFHS diet feeding. (D)

Volcano plot showing DEGs and (E) differential KEGG pathways induced by HFHS in WT-KCs. (**F-G**) DEGs in TFEB-KCs after HFHS diet feeding. (F) Volcano plot showing DEGs and (G) differential KEGG pathways induced by HFHS in TFEB-KCs. (**H**) Heatmap of KC2 signature identified by Blériot et al., 2021[28]. (**I**) Representative immunofluorescence images of TREM2^{+} CLEC4F^{+} KCs in HFHS diet-fed KC^{Cre} and KC^{Tfeb} male mice. Green: CLEC4F; red: TREM2; blue: DAPI. Scale bar = 50µm. (**J**) Mean fluorescent intensity (MFI) of CD36 in WT or TFEB-KCs quantified by flow cytometry (n = 2-7/group). FMO = Fluorescence minus one. Data represent individual biological replicates presented as means ±SEM. P-values for (J) were calculated using unpaired two-tailed Student's t-tests.

Supplemental Figure 5. Related to Main Figure 5.

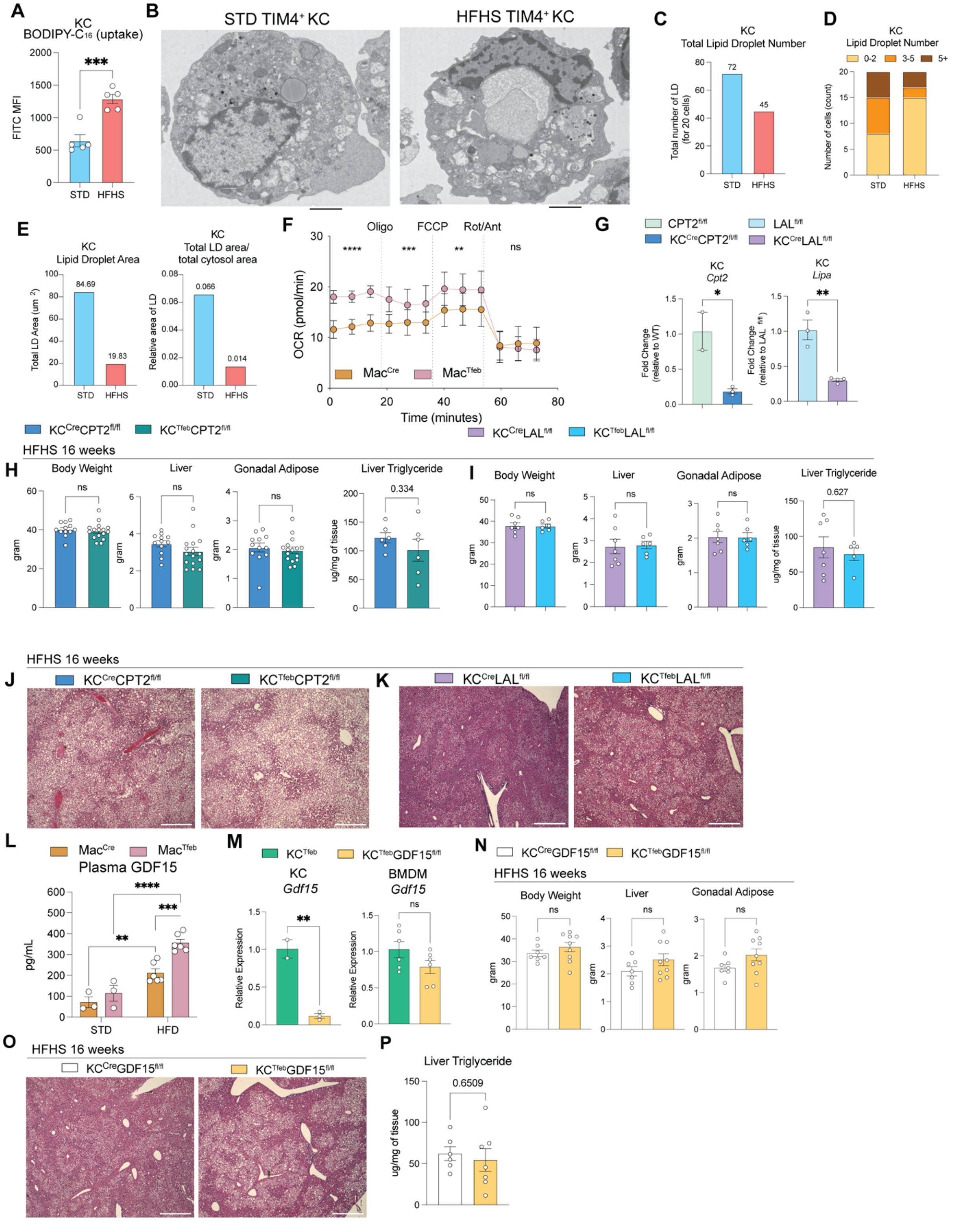

Supplemental Figure 5. Lipid accumulation and metabolic requirement in TFEB-KCs. (**A-D**) C57BL/6 male mice were fed STD or HFHS diet for 16 weeks. (A) MFI of BODIPY-C_{16} signal

in STD or HFHS KCs by flow cytometry after 1 minute incubation with FA (n = 5/group). (B) Representative electron micrographs of FACS-purified KCs, scalebar = 2 µm. (C) Total number of LDs, (D) distribution of cells with 0-2, 3-5, or 5+ LDs, (E) total LD area, and relative area of LD with respect to total cytosol area across 20 cells per condition. (**F**) Seahorse mitostress test on WT or TFEB-BMDMs. Oligo: oligomycin; Rot/Ant: rotenone/antimycin; OCR: oxygen consumption rate. (**G**) qPCR gene expression analyses of *Cpt2* and *Lipa (*encodes for LAL) in KCs isolated from respective mouse lines (n = 2-4/group). (**H-K**) Male mice fed 16-week HFHS diet. Body parameters, organ weights, and liver triglycerides of (H) $KC^{Cre}CPT2^{fl/fl}$ and $KC^{Tfeb}CPT2^{fl/fl}$ mice (n = 6-16/group), and (I) $KC^{Cre}LAL^{fl/fl}$ and $KC^{Tfeb}LAL^{fl/fl}$ (n = 5-7/group) (J-K) H&E images of livers from mice in fig. S5H and S5I . Scalebar = 500 µm. (**L**) Serum GDF15 measured by ELISA from Mac^{Cre} and Mac^{Tfeb} mice fed 16-week STD or high-fat diet (HFD) (n = 3-6/group). (**M**) qPCR gene expression analyses of *Gdf15* in KCs and BMDMs isolated from respective mouse lines (n = 2-3/group). (**N-P**) Male $KC^{Cre}GDF15^{fl/fl}$ and $KC^{Tfeb}GDF15^{fl/fl}$ were fed 16-week of HFHS diet (n = 7-9/group). (N) Body and organ weights of mice. (O) H&E images of livers. (P) Liver TAG measurement (n = 6-7/group). Data represents (A, G-I, L-N, P) individual biological replicates or (F) technical triplicates presented as means ±SEM. P-values were calculated using (A, F-I, M-N, P) unpaired two-tailed Student's t-tests and (L) two-way ANOVA followed by multiple t-tests. NS = not significant, *p < 0.05, **p < 0.01, ***p< 0.001, ****p<0.0001.

Supplemental Figure 6. Related to Main Figure 6.

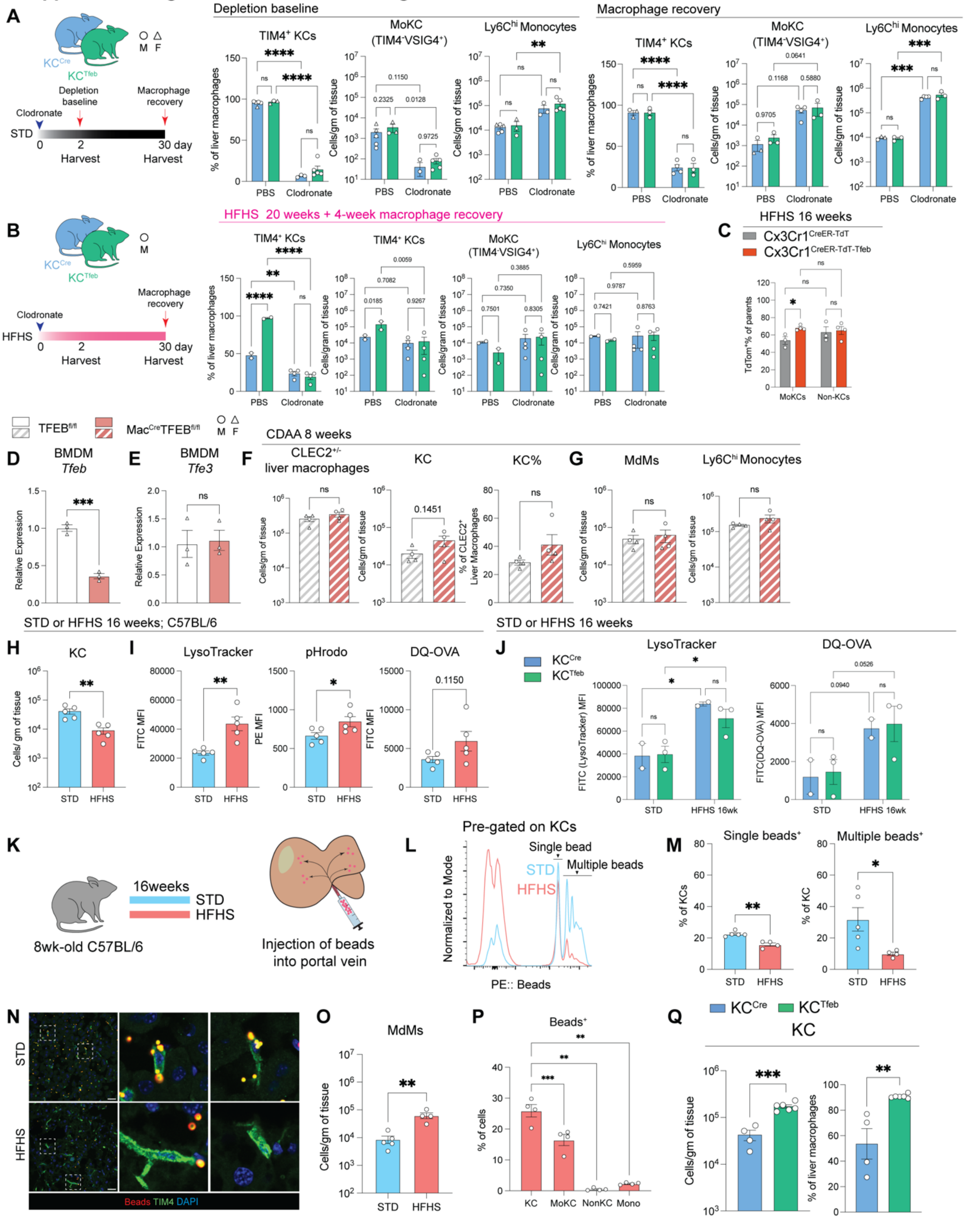

Supplemental Figure 6. Loss of TFEB in macrophages, and functional characterization of KCs during MASLD. (**A-B**) Liver macrophages/monocyte quantification by flow cytometry in

(A) STD-fed or (B) HFHS diet-fed KC^{Cre} and KC^{Tfeb} mice with clodronate-mediated macrophage depletion. (**C**) TdTomato reporter signal in MdM subsets in $Cx3cr1^{CreER\text{-}TdT}$ and $Cx3cr1^{CreER\text{-}TdT\text{-}Tfeb}$ mice, refers to Fig. 6D-F. (**D-G**) Control ($TFEB^{fl/fl}$) and LysM-Cre-driven TFEB-deficient mice ($Mac^{Cre}TFEB^{fl/fl}$) at homeostatic condition and after 8 weeks of CDAA diet. Circles represent males and triangles represent females. (D) qPCR gene expression analyses of *Tfeb* and (E) *Tfe3* in BMDMs. (F) Flow cytometric quantification of myeloid subsets, including KCs, (G) MdMs, and $Ly6C^{hi}$ monocytes (n = 4/group). (**H-I**) KCs were isolated from C56BL/6 male mice fed 16 weeks of STD or HFHS diet and incubated with various substrates to measure lysosomal activity (n = 5/group). (H) Flow cytometry quantification of KCs per gram of tissue. (I) MFI of lysotracker Green, pHrodo, and DQ-OVA in KCs. (**J**) MFI of Lysotracker Green and DQ-OVA in KCs isolated from KC^{Cre} and KC^{Tfeb} male mice fed 16 weeks of STD or HFHS diet (n = 2-3/group). (**K-P**) C57BL/6 male mice were fed STD or HFHS for 16 weeks and livers were *in situ* injected with fluorescent beads (n = 4-5/group). (K) Schematic of experiment. (L) Representative flow histogram of bead signal in KCs. (M) Percentage of KCs with single or multiple bead positive signals. (N) Representative immunofluorescence images of bead capturing in KCs. Red: beads; green: TIM4; blue: DAPI. Scale bar = 30µm. (O) Flow cytometric quantification of MdMs per gram of tissue. (P) Percentage of myeloid cells and monocytes with bead positive signal. (**Q**) Quantification of KCs per gram of tissue and as percentage in KC^{Cre} and KC^{Tfeb} mice used for in situ fluorescent bead assay. Refers to main Fig. 6M-P. Data represent individual biological replicates and are presented as means ±SEM. P-values were calculated using (A, B, C, J) two-way ANOVA followed by multiple t-tests, (D-H, M, O, Q) unpaired two-tailed Student's t-tests, and (P) one-way ANOVA followed by multiple t-tests. NS = not significant, $^*p < 0.05$, $^{**}p < 0.01$, $^{***}p < 0.001$, $^{****}p < 0.0001$.

Supplemental Figure 7. Related to Main Figure 7.

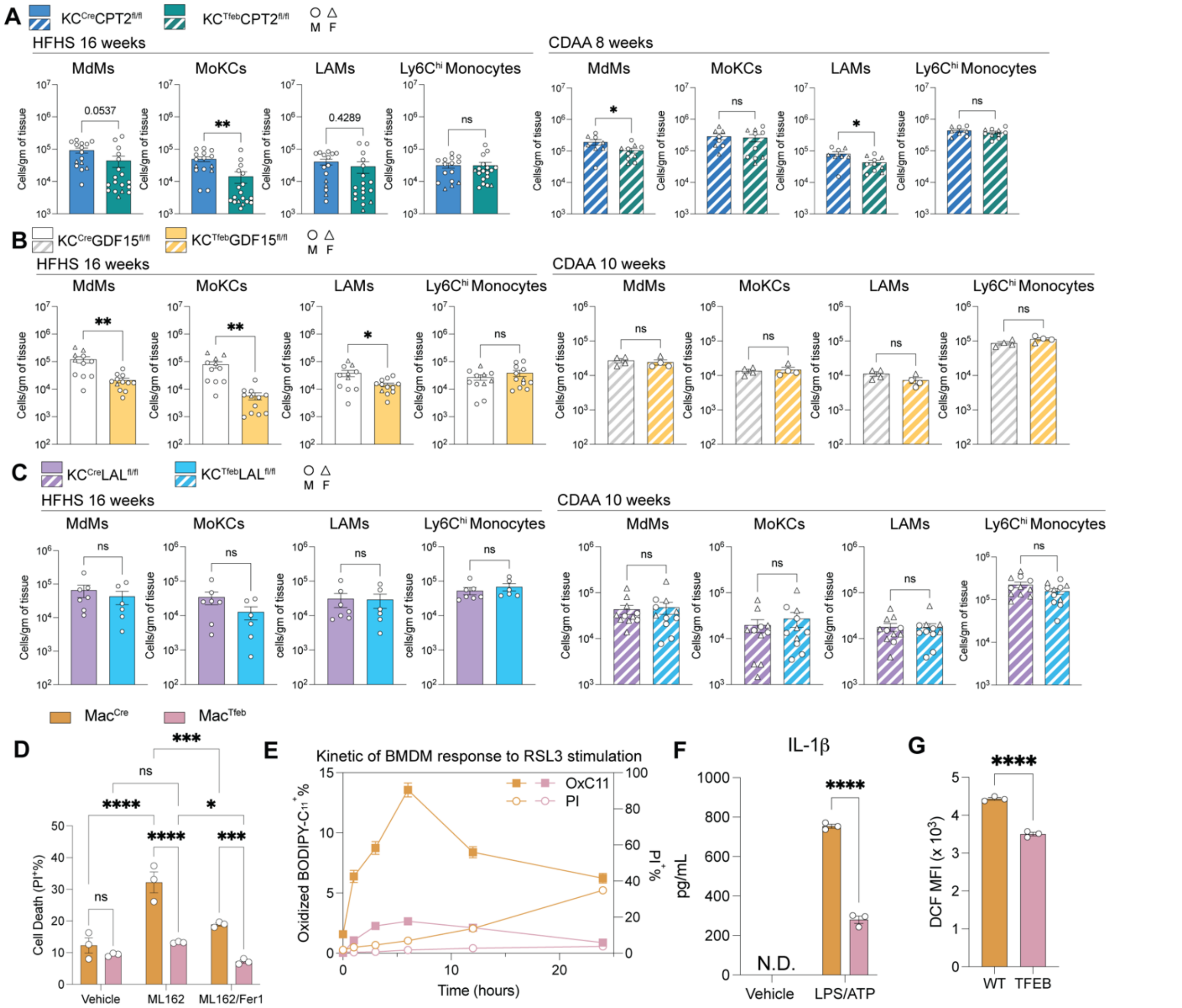

Supplemental Figure 7. Loss of MdMs in KC^{Tfeb} mice with additional knockout, macrophage cell death and gene expression analyses. (**A**) Flow cytometric quantification of myeloid and monocyte subsets in the livers of male and female KC^{Cre}CPT2$^{fl/fl}$ and KC^{Tfeb}CPT2$^{fl/fl}$ mice after 16 weeks of HFHS diet (n = 15-18/group) or 8 weeks of CDAA diet feeding (n = 8-10/group). (**B**) Flow cytometric quantification of myeloid and monocyte subsets in the livers of male and female KC^{Cre}GDF15$^{fl/fl}$ and KC^{Tfeb} GDF15$^{fl/fl}$ mice after 16 weeks of HFHS diet (n = 11-12/group) or 10 weeks of CDAA diet feeding (n = 4/group). (**C**) Flow cytometric quantification of myeloid and monocyte subsets in the livers of male and female KC^{Cre}LAL$^{fl/fl}$ and KC^{Tfeb} LAL$^{fl/fl}$ mice after 16 weeks of HFHS diet (n = 6-7/group) or 10 weeks of CDAA diet feeding (n = 11/group). (**D**) Ferroptotic death was measured by propidium iodide^{+} (PI^{+}) signal in WT- or TFEB-BMDMs treated with 5 μM ML162 ± 5 μM Fer1 for 2 h. (**E**) Flow cytometric percentage of PI^{+} and oxidized

BODIPY C11^{+} signal in cells treated with 5 μM RSL3 for 1 h, 3 h, 6 h, 12 h, and 24 h. Solid symbols represent oxidized BODIPY-C_{11} signal and open symbols represent PI staining. (**F**) Secreted IL-1β in WT- or TFEB-BMDMs stimulated with LPS and ATP. N.D. = not detectable. (**G**) DCF staining in WT- or TFEB-BMDMs. Data represents (A-C) biological replicate or (D-G) technical triplicates, and presented as means ±SEM. P-values were calculated using (A-C, F-G) unpaired two-tailed Student's t-tests, and (D) two-way ANOVA followed by multiple t-tests. NS = not significant, *$p < 0.05$, **$p < 0.01$, ***$p < 0.001$, ****$p < 0.0001$.

Supplemental Figure 8. Related to Main Figure 7.

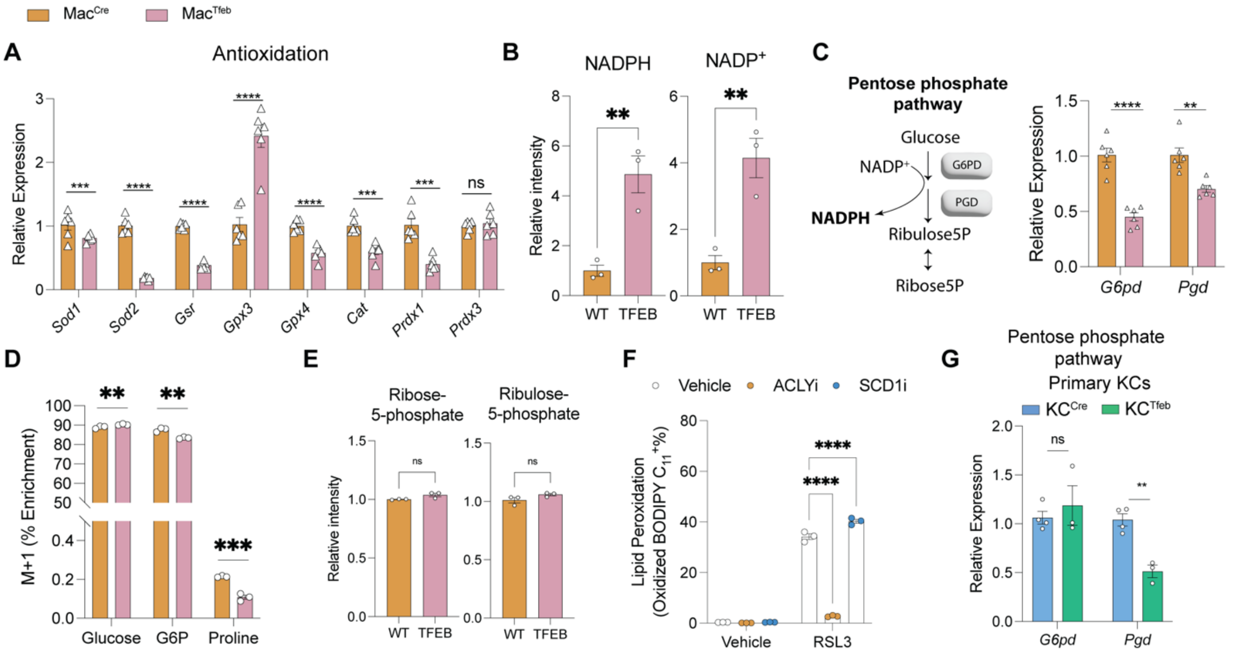

Supplemental Figure 8. Mechanism of NADPH accumulation in TFEB-macrophages. (**A**) Gene expression analyses on antioxidant enzymes in WT- or TFEB-BMDMs. (**B**) Relative intensity of NADPH and $NADP^+$ measured by mass spectrometry. (**C**) Schematic of pentose phosphate pathway (PPP) and relative expression of key PPP enzymes in WT- and TFEB-BMDMs. (**D**) Enrichment of molecules with M+1 isotope in WT and TFEB-BMDMs. (**E**) Relative intensity of PPP end products in WT- and TFEB-BMDMs measured by mass spectrometry. (**F**) Lipid peroxidation measured by oxidized BODIPY-C_{11} signal in WT-BMDMs pre-treated with ACLY inhibitor (BMS 303141) or SCD1 inhibitor (CAY10566) followed by co-treatment with RSL3 for 3 h. (**G**) qPCR analysis of PPP enzymes in primary KCs (n = 3-4/genotype). Data represents (A, C) biological duplicates with technical triplicates, (B, D-F) technical triplicates, or (G) biological replicates presented as means ± SEM. P-values were calculated using (A-E, G) unpaired two-tailed Student's t-tests, and (F) two-way ANOVA followed by multiple t-tests. NS = not significant, *p < 0.05, **p < 0.01, ***p< 0.001, ****p<0.0001.

Supplemental References

41. Daemen S, Chan MM, Schilling JD. Comprehensive analysis of liver macrophage composition by flow cytometry and immunofluorescence in murine NASH. *STAR Protocols*. 2021/06/18/ 2021;2(2):100511. doi:https://doi.org/10.1016/j.xpro.2021.100511

42. Dobin A, Davis CA, Schlesinger F, et al. STAR: ultrafast universal RNA-seq aligner. *Bioinformatics*. Jan 1 2013;29(1):15-21. doi:10.1093/bioinformatics/bts635

43. Liao Y, Smyth GK, Shi W. featureCounts: an efficient general purpose program for assigning sequence reads to genomic features. *Bioinformatics*. Apr 1 2014;30(7):923-30. doi:10.1093/bioinformatics/btt656
44. Patro R, Duggal G, Love MI, Irizarry RA, Kingsford C. Salmon provides fast and bias-aware quantification of transcript expression. *Nat Methods*. Apr 2017;14(4):417-419. doi:10.1038/nmeth.4197
45. Wang L, Wang S, Li W. RSeQC: quality control of RNA-seq experiments. *Bioinformatics*. Aug 15 2012;28(16):2184-5. doi:10.1093/bioinformatics/bts356
46. Robinson MD, McCarthy DJ, Smyth GK. edgeR: a Bioconductor package for differential expression analysis of digital gene expression data. *Bioinformatics*. Jan 1 2010;26(1):139-40. doi:10.1093/bioinformatics/btp616
47. Ritchie ME, Phipson B, Wu D, et al. limma powers differential expression analyses for RNA-sequencing and microarray studies. *Nucleic Acids Res*. Apr 20 2015;43(7):e47. doi:10.1093/nar/gkv007
48. Liu R, Holik AZ, Su S, et al. Why weight? Modelling sample and observational level variability improves power in RNA-seq analyses. *Nucleic Acids Res*. Sep 3 2015;43(15):e97. doi:10.1093/nar/gkv412
49. Kamburov A, Pentchev K, Galicka H, Wierling C, Lehrach H, Herwig R. ConsensusPathDB: toward a more complete picture of cell biology. *Nucleic Acids Res*. Jan 2011;39(Database issue):D712-7. doi:10.1093/nar/gkq1156
50. Kleverov M, Zenkova D, Kamenev V, Sablina M, Artyomov MN, Sergushichev AA. Phantasus, a web application for visual and interactive gene expression analysis. *Elife*. Jun 3 2024;13doi:10.7554/eLife.85722
51. Brunt EM, Kleiner DE, Wilson LA, Belt P, Neuschwander-Tetri BA, Network NCR. Nonalcoholic fatty liver disease (NAFLD) activity score and the histopathologic diagnosis in NAFLD: distinct clinicopathologic meanings. *Hepatology*. Mar 2011;53(3):810-20. doi:10.1002/hep.24127
